# Characterizing the MRI signature of hippocampal sclerosis of aging

**DOI:** 10.1186/s13195-026-02118-0

**Published:** 2026-06-22

**Authors:** Gustaf Rådman, Amanda E. Denning, Sadhana Ravikumar, Nicola Spotorno, Ranjit Ittyerah, Lisa M. Levorse, John L. Robinson, Theresa Schuck, Sydney A. Lim, Eunice Chung, Madigan Bedard, Winifred Trotman, Alejandra Bahena, María del Mar Arroyo-Jiménez, Alicia Vela, Esther Buendia, Maria Mercedes Iñiguez de Onzoño Martin, María Pilar Marcos Rabal, Mónica Muñoz-López, Rosanna K. Olsen, John A. Detre, Edward B. Lee, David A. Wolk, Daniel T. Ohm, Corey T. McMillan, Ricardo Insausti, David J. Irwin, Paul A. Yushkevich, Laura E. M. Wisse, the Alzheimer’s Disease Neuroimaging Initiative

**Affiliations:** 1 Department of Clinical Sciences Lund, Lund University, BMC I12, Lund, 221 84 Sweden; 2 Department of Radiology, University of Pennsylvania, 3400 Spruce Street, Philadelphia, PA 19104 USA; 3 Department of Clinical Sciences Malmö, Lund University, Box 50332, Malmö, Lund, 202 13 Sweden; 4 Department of Pathology and Laboratory Medicine, University of Pennsylvania, 3400 Spruce Street, Philadelphia, PA 19104 USA; 5 Department of Neurology, University of Pennsylvania, 3400 Spruce Street, Philadelphia, PA 19104 USA; 6 Human Neuroanatomy Laboratory, Neuromax CSIC Associated Unit, University of Castilla-La Mancha and Institute for Biomedicine, Plaza de La Universidad, 2. Edificio José Prats, Albacete, 02071 Spain; 7 Department of Psychology, University of Toronto, 100 St. George Street, Toronto, ON M5S 3G3 Canada; 8 Rotman Research Institute, Baycrest Academy for Research and Education, 3560 Bathurst Street, Toronto, ON M6A2E1 Canada

**Keywords:** Hippocampal sclerosis of aging, Magnetic resonance imaging, Neurodegenerative disease, Alzheimer’s Disease neuropathologic change, Dementia

## Abstract

**Background:**

Hippocampal sclerosis of aging (HS-A), a common cause of dementia, can currently only be diagnosed at autopsy. We aimed to identify and evaluate MRI metrics to distinguish HS-A from Alzheimer’s Disease neuropathologic change (ADNC) and cases with limited/no pathology.

**Methods:**

HS-A (N = 5), ADNC (N = 10), and limited/no pathology (N = 12) cases were compared on postmortem MRI signatures: manually measured cornu ammonis (CA)1/subiculum thickness, grey matter signal intensity, and automated hippocampal subfield thickness metrics. Similar metrics were obtained in T_1_-weighted antemortem MRI in an initial dataset (HS-A = 4, ADNC = 7, limited/no pathology = 25) for group differences and discrimination (HS-A vs ADNC). T_1_-weighted metrics were then evaluated in a second dataset (HS-A = 6, ADNC = 18) and in a pooled post-hoc analysis combining HS-A and ADNC cases from both datasets (N_HS-A_ = 10, N_ADNC_ = 25).

**Results:**

Postmortem MRI showed hippocampal thinning and grey matter hypointensity in HS-A at the CA1-subiculum junction more severe than in ADNC and limited/no pathology cases. In antemortem MRI, differentiating HS-A and ADNC based on anterior/posterior manual measures of CA1/subiculum thickness and hippocampal volumes displayed good discrimination in dataset 1, but lower discriminative performance in dataset 2. In complementary analyses pooling both datasets and adjusting for age, manual thickness achieved good performance (area under the curve (AUC) = 0.80–0.87), while anterior, posterior, and whole hippocampal volumes showed excellent discrimination (AUC = 0.94–0.98).

**Limitations:**

The study included a relatively small and neuropathologically heterogeneous sample. The final antemortem analyses were exploratory, reflecting challenges in replicating findings across independent datasets. Classification was limited to HS-A and ADNC, leaving it uncertain how the metrics perform when comparing HS-A with other diagnostic groups. HS-A diagnoses were determined postmortem, often several years after MRI, and most measures relied on standard imaging sequences with limited resolution to assess fine-grained hippocampal subfields.

**Conclusions:**

HS-A displays distinct changes in the hippocampus that are detectable through structural MRI. Associated quantifiable MRI metrics may serve as promising tools in aiding antemortem HS-A diagnosis but require further validation in larger cohorts and against other dementia-related diseases.

**Supplementary Information:**

The online version contains supplementary material available at https://doi.org/10.1186/s13195-026-02118-0.

## Background

Hippocampal sclerosis of aging (HS-A) is defined by neuronal loss and gliosis in hippocampal subfields cornu ammonis 1 (CA1) and subiculum that is disproportional to tau pathology in the same regions [1, 2]. HS-A is suggested to be a late-stage phenomenon developed in a subset of cases with TAR DNA-binding protein 43 (TDP-43) proteinopathy [3, 4] and most notably limbic predominant age-related TDP-43 encephalopathy (LATE) [5–7]. However, it can also occur in the context of cerebrovascular diseases [8, 9]. Cognitively, HS-A with or without TDP-43 pathology has been linked to increased odds of dementia [3] and to deficits across several cognitive domains [2, 3, 10, 11] with a particular emphasis on progressive memory decline [12]. As such, HS-A is often described as a mimic of amnestic Alzheimer’s Disease (AD) [12–14]. A notable difference to AD is that HS-A primarily affects the oldest old [2, 15] with estimates of prevalence among adults above the age of 80 ranging between 10 and 25% [16–18].

To date there are no established in vivo biomarkers for HS-A meaning that confirmation of HS-A is only possible through autopsy. This poses challenges for both in vivo dementia research and clinical practice. Magnetic resonance imaging (MRI) is a potential tool to identify HS-A in-vivo. Studies of HS-A utilizing MRI consistently find smaller hippocampal volumes compared to both cognitively normal and cases with AD or LATE without concomitant HS-A, differences detectable up to a decade before autopsy [19–23]. However, longitudinal studies have found slightly slower atrophy rates among HS-A than AD [21, 22]. On one hand, this could suggest that HS-A atrophy has a relatively slow trajectory. Alternatively, longitudinal studies may have assessed HS-A atrophy at a stage when the most pronounced neurodegeneration had already occurred.

The identification of HS-A-related changes on structural MRI has led several studies to suggest its potential utility for in vivo diagnosis [20, 23, 24]. However, efforts to systematically characterize the specific MRI signatures of HS-A, which is crucial to distinguish it from other dementia-related disorders that also involve hippocampal atrophy, have been limited. A more granular analysis of the imaging signature of HS-A may therefore yield clinically valuable assessment tools.

In this study, we aimed to characterize MRI signatures of HS-A and evaluate their diagnostic performance, focused here on the differentiation between HS-A and AD. First, we examined ultra-high-resolution postmortem MRI from subjects with neuropathologically confirmed HS-A, AD neuropathologic change (ADNC), and cases with limited to no pathology, obtained from brain donations to the Human Neuroanatomy Lab (HNL) in Albacete, Spain, and the University of Pennsylvania's Center for Neurodegenerative Disease Research (CNDR). We then quantified the observed MRI features across the three groups using both focal manual measures targeting the most severely affected region and global automated measurements utilizing an in-house developed postmortem segmentation algorithm. We expected to find selective atrophy in CA1 and subiculum most discriminant of groups. Next, we translated these findings to antemortem MRI, comparing similar focal and global hippocampal measurements between neuropathologically confirmed HS-A, ADNC cases, and healthy controls. Finally, we evaluated our findings in a second dataset with antemortem MRI in cases with neuropathologically confirmed HS-A and ADNC from the Alzheimer’s Disease Neuroimaging Initiative database (ADNI).

## Methods

### Cohorts

#### Postmortem dataset

We reviewed all brain donations with postmortem 9.4T MRI and histology data available from CNDR and HNL. Flowchart depicting inclusion and exclusion of cases is available in Supplementary Material (Figure S1). Classification of HS-A, defined as near-complete or complete neuron loss in CA1 and subiculum, was initially made using hematoxylin and eosin staining in the hemisphere contralateral to postmortem MRI. To ensure diagnosis in the scanned hemisphere, Nissl-stained slices were used to confirm HS-A in the hemisphere from which we obtained the postmortem MRI. In the hemisphere contralateral to postmortem MRI, neuropathologists at CNDR applied current diagnostic criteria [25] to assign Thal phases [26], Braak stages [27] and CERAD scoring for neuritic plaques [28]. Semiquantitative ratings for the presence of TDP-43 or α-synuclein proteinopathy in MTL areas were also obtained and used for the selection of cases, details on which are available in previous publications [29, 30]. In brief, pS409/410 was used to detect phosphorylated TDP-43 deposits, and Syn303 to detect the presence of pathological conformation of α-syn and based on these each region was assigned a semiquantitative score rated as none (0), rare (0.5), mild (1), moderate (2), or severe (3) for individual lesions.

Based on these evaluations, cases were assigned to one of three groups. For the HS-A group, we included all cases with a diagnosis of HS-A from the hemisphere contralateral to MRI and confirmation of HS-A in the ipsilateral hemisphere. Cases were included irrespective of comorbid pathologies since cases of pure HS-A are rare [15, 31], resulting in a total of six cases. For the group with ADNC, we included cases with intermediate to high ADNC but no HS-A and rare or no TDP-43 pathology in the medial temporal lobe (0.5 or 0 on the semiquantitative scale). Excluding those with TDP-43 pathology was intended to reduce the chance of HS-A in the contralateral hemisphere with postmortem MRI available. These criteria resulted in a total of 10 ADNC cases. For the group with limited to no pathology, cases were included that had no HS-A and limited or no other neuropathology likely to affect the hippocampus, including α-synuclein pathology, TDP-43 pathology or ADNC. The age of limited/no pathology cases was restricted to the range of the patient cohorts to avoid conflating the effects of age and disease on volumetric estimates, resulting in the exclusion of three cases who were substantially younger than the remaining cohort (age range for excluded cases: 57–61; age range of remaining sample: 68—99) resulting in 12 limited/no pathology cases. Demographic, clinical and neuropathological information for the full set of cases is available in Table 1.

**Table 1:** Demographic information across all datasets

	**Postmortem dataset**					**Antemortem dataset 1**					**Antemortem dataset 2**			
	**N**	**HS-A** **Mean ± SD**	**ADNC** **Mean ± SD**	**Control** **Mean ± SD**	***p-*value**	**N**	**HS-A** **Mean ± SD**	**ADNC** **Mean ± SD**	**Control** **Mean ± SD**	***p-*value**	**N**	**HS-A** **Mean ± SD**	**ADNC** **Mean ± SD**	***p-*value**
Demographic variables														
N	28	6	10	12		29	4	7	18		26	6	18	
Age at MRI*	28	84.5 ± 8.12	76.9 ± 5.40	80.7 ± 10.6	0.239	29	74.5 ± 13.7	73.9 ± 4.22	73.6 ± 4.58	0.967	26	84.7 ± 3.44	84.9 ± 4.42	0.901
Sex (%Male)	28	4 (66.7)	7 (70)	8 (66.7)	1.000	29	2 (50.0)	4 (57.1)	5 (27.8)	0.408	26	6 (100)	13 (72.2)	0.280
Years of education (N)	18	17.0 ± 3.03 (6)	18.2 ± 1.48 (10)	14.5 ± 4.95 (2)	0.168	29	18.0 ± 1.63 (4)	18.3 ± 1.38 (7)	17.9 ± 1.95 (18)	0.913	26	16.7 ± 1.21 (6)	16.2 ± 2.10 (18)	0.535
Clinical variables														
CDR – Global Score (N)	15	2.75 ± 0.50 (5)	1.61 ± 0.93 (8)	0.25 ± 0.35 (2)	**0.011**	26	2.50 ± 0.71 (2)	1.83 ± 0.98 (6)	0.00 ± 0.00 (18)	** < 0.001**	26	1.00 ± 0.55 (6)	1.11 ± 0.93 (18)	0.728
CDR – Years from MRI (N)	15	−1.61 ± 1.36 (5)	−1.17 ± 0.90 (8)	−1.96 ± 1.06 (2)	0.571	26	0.71 ± 1.00 (2)	1.40 ± 3.16 (6)	−0.07 ± 0.09 (18)	0.127	26	0.09 ± 0.21 (6)	−0.01 ± 0.03 (18)	0.280
MMSE – Total Score (N)	17	19.0 ± 5.66 (6)	20 ± 9.37 (9)	28.5 ± 2.12 (2)	0.342	28	13.0 ± 9.83 (4)	16.5 ± 9.79 (6)	29.7 ± 0.57 (18)	** < 0.001**	26	22.3 ± 2.16 (6)	19.9 ± 7.49 (18)	0.239
MMSE – Years from MRI (N)	17	−5.99 ± 2.93 (6)	−2.49 ± 2.34 (9)	−6.88 ± 0.36 (2)	**0.025**	28	−0.25 ± 0.80 (4)	1.20 ± 1.09 (6)	−0.07 ± 0.09 (18)	** < 0.001**	26	0.10 ± 0.21 (6)	−0.06 ± 0.22 (18)	0.150
Cognitively impaired (%) (N)	18	6 (100) (6)	10 (100) (10)	0 (0) (2)	**0.007**	29	4 (100) (4)	7 (100) (6)	0 (0) (18)	** < 0.001**	26	6 (100) (6)	18 (100) (18)	-
Neuropathology														
Intermediate to high AD (%)	28	1 (16.7)	10 (100)	0 (0)	** < 0.001**	11	0 (0)	7 (100)	-	** < 0.001**	26	4 (66.7)	18 (100)	0.054
A-score	27†	1.33 ± 1.03	2.70 ± 0.48	0.92 ± 0.29	** < 0.001**	11	0.50 ± 0.58	2.71 ± 0.49	-	** < 0.001**	26	2.00 ± 0.63	2.61 ± 0.50	0.067
B-score	28	1.33 ± 0.52	2.50 ± 0.53	1.18 ± 0.60	** < 0.001**	11	1.00 ± 0.82	2.57 ± 0.53	-	**0.004**	26	2.33 ± 1.03	2.78 ± 0.43	0.348
C-score	28	0.67 ± 1.03	2.40 ± 0.52	0.83 ± 0.39	**0.002**	11	0.00 ± 0	2.57 ± 0.79	-	** < 0.001**	26	2.33 ± 1.03	2.22 ± 1.06	0.826
FTLD-TDP (%)	28	3 (50)	0 (0)	0 (0)	**0.006**	11	3 (75)	0 (0)	-	**0.001**	26	1 (16.7)	0 (0)	0.25
Lewy body disease (%)	28	1 (16.7)	5 (50)	0 (0)	**0.012**	11	0 (0)	4 (57.1)	-	**0.001**	26	0 (0)	5 (27.8)	0.289
LATE-NC (%)	28	3 (50)	0 (0)	0 (0)	**0.006**	11	1 (25)	0 (0)	-	0.111	26	5 (83.3)	0 (0)	** < 0.001**
PART (%)	28	1 (16.7)	0 (0)	0 (0)	0.214	11	1 (25)	0 (0)	-	0.111	26	1 (14.3)	0 (0)	0.250

Continuous variables are presented as mean and standard deviation with group differences assessed by analysis of variance (Postmortem dataset and Antemortem dataset 1) or t-test (Antemortem dataset 2). Categorical variables are presented as numbers and percentages and group differences assessed by Chi-square tests. Statistically significant differences after multiple comparison correction using FDR are marked in bold

*Abbreviations*: *CDR *Clinical Dementia Rating, *MMSE *Mini Mental State Examination, *FTLD-TDP *Frontotemporal lobar degeneration with TDP proteinopathy, *LATE-NC *Limbic-predominant age-related TDP-43 encephalopathy neuropathologic change, *PART *Primary age-related tauopathy, *HS-A *Hippocampal sclerosis of aging, *ADNC *Alzheimer’s Disease neuropathologic change

^*^Age at MRI in postmortem MRI dataset indicates age at autopsy

^†^Amyloid values in hippocampus were inconclusive for one limited/no pathology case. Braak stage = 0, CERAD score = 2. Neuropathologist final judgement: low level ADNC

#### Antemortem dataset 1

Flowchart depicting inclusion and exclusion of cases is available in Supplementary Material (Figure S1 and Figure S2). For HS-A cases, we included all autopsy-confirmed cases with both T_2_-weighted (T_2_) and T_1_-weighted (T_1_) antemortem MRI available, including one case removed from the postmortem MRI dataset due to unilateral HS-A in the hemisphere without postmortem MRI. Antemortem analyses were restricted to the hemisphere with confirmed HS-A. As an additional criterion, since confirmation of HS-A relies on a neuropathological assessment, we limited the antemortem interval between scan and death to five years, leading to two exclusions. This resulted in four cases assigned to the HS-A group.

For ADNC cases, we ensured available antemortem scans were acquired within the same year or at a point in time after clinical diagnosis of probable AD dementia to increase the chance that any changes observed on MRI are attributable to ADNC. This resulted in eight cases assigned to the ADNC group. As with HS-A cases, only the hemispheres with relevant neuropathological assessments were analyzed.

Since too few cases with low to no neuropathology from CNDR and HNL had antemortem MRI available, we formed a new limited/no pathology group from amyloid-beta negative cognitively normal cases from ADNI (https://adni.loni.usc.edu/). The ADNI was launched in 2003 as a public–private partnership, led by Principal Investigator Michael W. Weiner, MD. The primary goal of ADNI has been to test whether serial magnetic resonance imaging (MRI), positron emission tomography (PET), other biological markers, and clinical and neuropsychological assessment can be combined to measure the progression of mild cognitive impairment (MCI) and early Alzheimer’s disease (AD).

In control cases from ADNI, amyloid-beta status was determined using 18F-florbetapir (AV45) or 18F-florbetaben (FBB) positron emission tomography (PET), where standardized uptake value ratios (SUVRs) were calculated for a cortical summary measure (frontal, anterior cingulate, precuneus, and parietal cortex) relative to the whole cerebellum. Amyloid-beta positivity cut-off values used were 1.11 for AV45 and 1.08 for FBB, respectively [32, 33]. To ensure the absence of neurodegenerative diseases at the time of MRI to the best extent possible, these individuals had to be amyloid-beta negative at least two years following the evaluated MRI. We also excluded cases based on ptau217 values using the Fujirebio Lumipulse G1200 automated immunoassay, where a value above 0.128 pg/mL was classified as intermediate levels [34, 35]. Here, we required all to have below intermediate levels at least 2 years following MRI. Finally, we excluded cases with positive α-synuclein seeding activity as determined by the Amprion α-synuclein seed amplification assay [36]. See flowcharts in Supplementary Materials (Figure S1 and S2) for specific exclusions for each of the biomarkers. Finally, these individuals had to be free from a clinical diagnosis of mild cognitive impairment or dementia for at least two years following MRI. Total sample used in the limited/no pathology group was N = 18. Demographic, clinical and neuropathological information is available in Table 1.

#### Antemortem dataset 2

We next aimed to validate the antemortem analyses in a separate dataset fully consisting of ADNI cases. Flowchart depicting inclusion and exclusion of cases is available in Supplementary Material (Figure S2). HS-A and ADNC cases were selected from the subset of cases in ADNI with neuropathological assessment and antemortem MRI available. For HS-A cases this included autopsy-verified HS-A with a maximum scan-autopsy interval of five years and with clinical diagnosis of dementia at the time of the scan. ADNC cases had to have intermediate to high ADNC, no confirmed HS-A, and no to low TDP-43 pathology in amygdala, hippocampus or entorhinal cortex at autopsy. Both groups also had to have clinical diagnosis of dementia at the time of the scan. For both patient groups, we restricted our analysis to the hemisphere with neuropathological evaluation. Finally, we only included cases with data available across all assessed metrics, which led to the exclusion of one ADNC case and one HS-A case from dataset 2 with failed automated segmentations. In total, this resulted in six cases in the HS-A group and eighteen cases in the ADNC group. Demographic, clinical and neuropathological information is available in Table 1.

#### Ethical considerations

Human brain specimens were obtained in accordance with the University of Pennsylvania Institutional Review Board guidelines, and the Ethical Committee of UCLM. If possible, preconsent during life was given, and in all other cases next-of-kin consent at death was given. Antemortem imaging was approved by the Institutional Review Board of the University of Pennsylvania and informed consent was provided by all subjects. For ADNI, the study was approved independently at each site by a local review board and subjects provided informed consent prior to participation.

### Histology and MRI acquisition

#### Postmortem dataset

In specimens from CNDR, hemispheres were fixed in 10% formalin solution for at least 30 days before dissection to selectively remove the temporal lobe. In specimens from HNL, cases were fixed by perfusion with 4% paraformaldehyde through both carotid arteries for 24–48 h, after which the brain was extracted and postfixed in 4% paraformaldehyde [37]. The excised temporal lobe was then imaged on a Varian 9.4 T 30 mm horizontal bore animal scanner at a 200 × 200 × 200 µm^3^ resolution overnight using a T_2_ sequence. For a more detailed description of the procedure involved in both histology and MRI acquisition, please see previous publications [38, 39].

#### Antemortem datasets

##### Antemortem dataset 1

We utilized both high resolution T_2_ coronal scans and T_1_ scans acquired at 3 tesla MRI. Both sequences were available in all evaluated cases. T_2_ images were acquired using turbo spin echo sequences and acquired perpendicular to the long axis of hippocampus with a limited field of view centered on the medial temporal lobe (in-plane resolution 0.4 × 0.4 mm, slice thickness CNDR: 1.2–3 mm, ADNI: 2 mm). This sequence is common in neuroanatomical research on hippocampal subfields since it allows good visualization of important landmarks of hippocampal anatomy [40]. T_1_ images from CNDR subjects were acquired using a magnetization-prepared rapid acquisition gradient recalled echo (MPRAGE) sequence with 0.8 or 1.0 mm^3^ isotropic voxels. T_1_ images from ADNI were either acquired using an MPRAGE sequence or an inversion recovery fast spoiled gradient recalled echo (IR-FSPGR) sequence. The MRI protocols used to acquire the T_1_ MRI scans in ADNI have been previously described in Jack et al. [41].

##### Antemortem dataset 2

The second dataset consisted of only T_1_ scans, as T_2_ scans were not available in ADNI subjects with neuropathology data. The T_1_ images of these cases were acquired using the same parameters as described above.

### MRI Measures of hippocampal structure and grey/white matter intensity ratio

#### Postmortem dataset

Manual measurements were performed using ITK-SNAP [42] on slices across the anterior, middle and posterior hippocampus following a protocol developed for this study. Each of the manually extracted measurements are also exemplified in Figure S3 of the Supplementary material. For the anterior hippocampus, we extracted manual measurements at the most posterior slice in which the dentate gyrus covers the full width of the hippocampus. For the middle hippocampus, we performed our measurements 5 mm (± 0.6 mm) posterior to the posterior tip of the uncus. For the posterior hippocampus, we performed our measurements 8 mm (± 0.6 mm) anterior to the posterior tip of the tail. Within slices, measurement locations were determined by qualitative assessment of the visible features of HS-A across all evaluated specimen, aiming to capture the most severely affected area (see Section “Characterization of the MRI signature of HS-A”). Based on our observations, we sampled a location close to the border of cornu ammonis 1 (CA1) and subiculum covering approximately 1.5 mm in width. The location of this border was approximated based on anatomical knowledge and published atlases [43, 44]. For manual measurement of thickness, measures were performed on a single slice but at three separate, adjacent locations and then averaged together. We also extracted measures of grey matter (GM) and white matter (WM) voxel intensity which were compiled into a GM/WM intensity ratio. These were extracted as close to each other as possible within each slice (while avoiding partial volume voxels) and repeated across three consecutive slices. Aiming to sample 1.5 mm in a lateral-medial direction, two rows of 8 voxels were selected for each tissue type and slice, alternatively when two rows of voxels were not available 16 adjacent voxels were sampled.

We next segmented hippocampal subfields and associated white matter regions using an in-house developed automated algorithm for postmortem MRI, further details provided in previous publications [39, 45]. In brief, this algorithm leverages an ex vivo MRI atlas of the medial temporal lobe, with subregions labels defined based on cytoarchitectural features derived from serial histology images. For each case, the atlas was registered to the postmortem MRI to map the hippocampal subfield labels to individual subject space. Average whole subfield thickness was extracted for CA1-3, stratum radiatum, lacunosum and moleculare (SRLM), subiculum, pre- and para-subiculum and the perforant pathway (defined as the white matter of the subicular regions). We also obtained measures of volume for dentate gyrus due to its amorphous structural shape not lending itself well to measures of thickness. Pre- and para-subiculum measures of thickness were not available for one HS-A case and dentate gyrus volume measures were not available for one HS-A case.

#### Antemortem datasets

Due to limited visibility of important landmarks and much more heterogenous morphology at the most posterior end of hippocampus, we limited the antemortem manual measurements to only the anterior hippocampus and middle/posterior hippocampus (corresponding to the middle location in postmortem MRI). Each of the manually extracted measurements are exemplified in Figure S1 of the Supplementary. For the anterior hippocampus, measurements were made on the most anterior slice where the full width of dentate gyrus is visible. For the middle/posterior hippocampus, measurements were made approximately 5 mm posterior to the uncus on a slice with sufficient image quality and landmark visibility.

In T_2_ MRI, we extracted thickness in the exact same manner as in postmortem MRI. Due to poor image quality, manual measurement of thickness could not be performed in one ADNC case and one limited/no pathology case, and it was limited to one hemisphere in another limited/no pathology case. For GM/WM intensity, due to larger voxel sizes, we used a total of 8 voxels per slice across three slices for anterior hippocampus, and three slices for middle/posterior hippocampus. Manual extraction of GM/WM intensity could not be performed in one limited/no pathology case due to image quality. We also utilized Automatic Segmentation of Hippocampal Subfields (ASHS; [46]) to segment hippocampal subfields and derive CA1, CA2, CA3, dentate gyrus and subiculum volumes. Segmentations were made using the Penn ABC-3T atlas [47] based on the segmentation protocol by Berron et al. [48]. Quality assessment was performed for all segmentations, but no corrections were applied beyond exclusion of cases with complete segmentation failure to mimic a clinical setting where editing segmentations would be prohibitive. Three limited/no pathology cases were excluded due to complete segmentation failure, defined as ASHS not terminating successfully or the segmented region being entirely (or predominantly) outside the intended area.

**Fig. 1:**
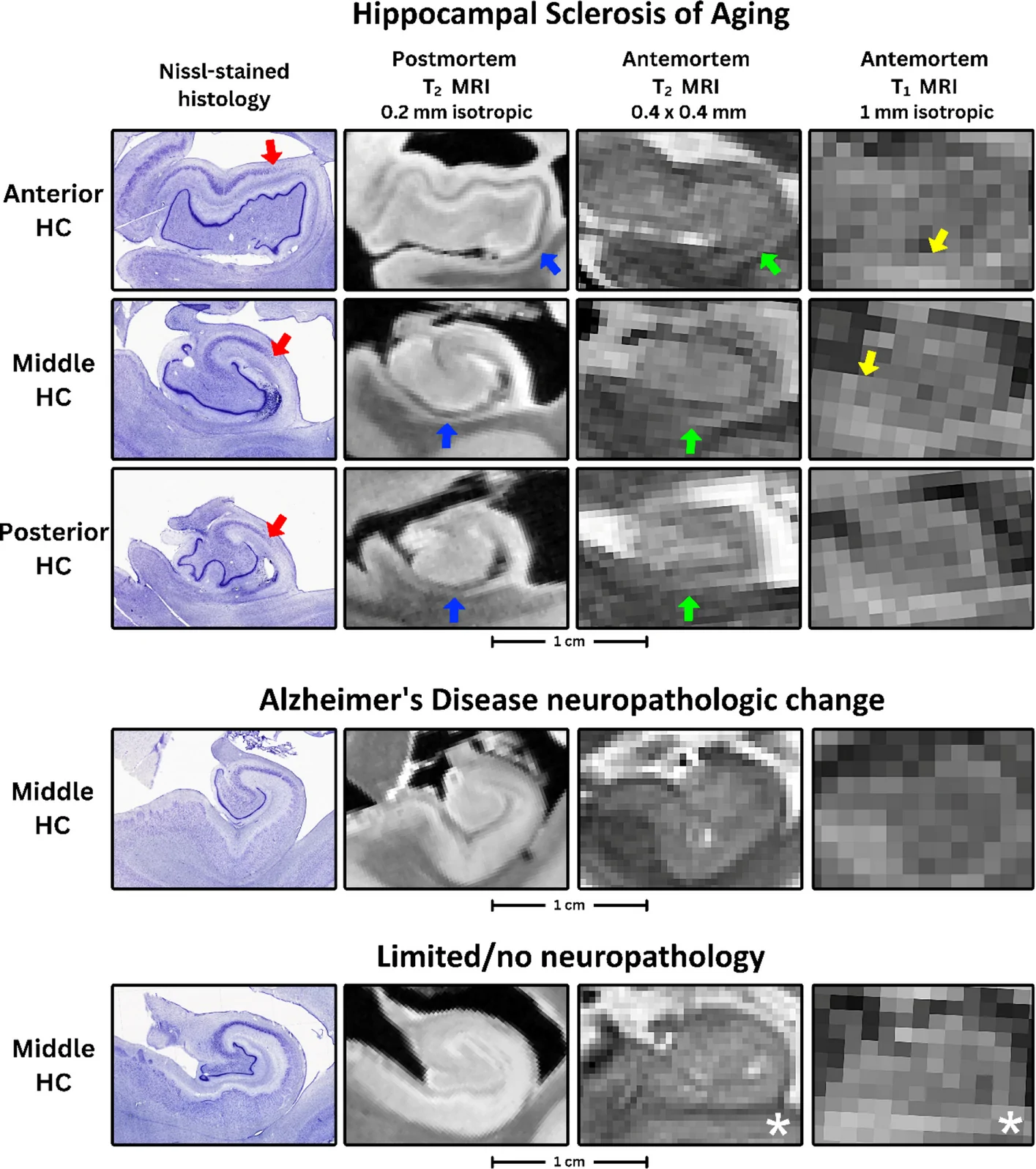
Example HS-A specimen (Male, 83) in Nissl-stained histology, postmortem MRI and in antemortem T_2_ and T_1_ MRI across three sections of the hippocampus (HC). For comparison, middle hippocampus is depicted for one case with ADNC (Male, 84) and one with limited/no pathology (histology and postmortem MRI: Female, 85; antemortem MRI T_2_ and T_1_: Female, 83). Images are taken from the same subject and approximate the same location with the exception of limited/no pathology cases in antemortem T_2_ and T_1_ (marked with star) as a separate pool of subjects was used antemortem. In Nissl-stained histology, pyramidal layer neuron loss is clearly visible extending across subfields CA1 and subiculum. Red arrows mark the approximate start of severe neuron loss, which in the example extend across the full extent of CA1 and subiculum. In postmortem MRI, cortical thinning and hypointense grey matter signal (blue arrows) can be clearly seen consistently across the evaluated specimen with a centerpoint near the border of CA1 and subiculum. This location could therefore be useful for focal measurement of HS-A-specific MRI changes. In T_2_ MRI antemortem MRI, thinning of CA1 and subiculum is visible across most slices while changes to intensity are less visible. The same location as in postmortem MRI can be used to sample more focal measures (green arrows). In T_1_ MRI both features are not visible, but nevertheless might be identified using a quantitative approach. However, limited SRLM visibility required shifting the measured region (yellow arrows)

In T_1_ MRI, limited visibility of the SRLM complicates manual extraction of thickness for hippocampal subfields [40]. To circumnavigate this issue, we used alternative measurement strategies to maximize consistency. In the anterior hippocampus, we anchored our thickness measure to the most anterior appearance of the hippocampal fissure, visible as hypointense voxels, which we could reliably identify in T_1_ MRI. The slice in the anterior hippocampus was selected at a location with maximal visibility of this landmark, which was fairly consistent between cases. While at a slightly different location from the postmortem measurements, this location gives an estimation of thickness and gray/white matter hyperintensity ratio in a region roughly corresponding to the middle/lateral subiculum, being a bit more medial but not far from the border with CA1 where measures were performed on postmortem MRI. In the middle/posterior hippocampus we moved our extraction to the most medial point of the dentate gyrus and measured cortical thickness anchored to the cerebrospinal fluid of the ambient cistern (see Fig. 1). This location reflects a medial portion of the subiculum proper and therefore deviates from the most optimal location highlighted in our postmortem analysis but ultimately allowed us to obtain thickness estimates despite limited visibility of the SRLM. For GM/WM intensity ratios we followed the same protocol as in T_2_ MRI, namely 8 voxels on each slice for GM and WM respectively on three slices in both the anterior and middle/posterior hippocampus. Finally, we utilized the ASHS-T_1_ atlas [49] to obtain automated segmentations of anterior and posterior hippocampal volumes. Quality assessment was performed for all segmentations, again avoiding manual correction to mimic a clinical setting where editing segmentations would be prohibitive. One case from the HS-A group (dataset 2), one from the AD group (dataset 2) and five limited/no pathology cases were excluded due to complete segmentation failure.

To minimize the risk of bias in manual tracing, the tracer was blinded to group designation during manual thickness extraction. In the case of postmortem MRI, blinding was not applied, as the presence of HS-A was visually unmistakable, rendering blinding ineffective.

For all subjects, we also calculated total intracranial volume for adjustment of volume measures. Specifically, Estimated Total Intracranial Volume (eTIV) values were derived using FreeSurfer (HNL/CNDR: version 7.4.1; ADNI: version 6.0) [50].

#### Statistical analyses

In limited/no pathology cases both hemispheres were used for all cases and were averaged, whereas for ADNC and HS-A cases only the hemispheres on which neuropathological assessment was performed were analyzed. Group comparisons were conducted using the Mann–Whitney U test, given the limited sample size and non-normal distribution of the measures. Effect sizes were calculated using a rank-biserial correlation coefficients [51]. All statistical tests were adjusted for multiple comparisons using false discovery rates (FDR), applied separately to each set of measurements.

All antemortem volume measures were adjusted for eTIV using a regression-based approach. Specifically, the linear relationship between the volume of interest and eTIV was first estimated within the limited/no pathology group to define the normal scaling factor (β). This slope was then applied to the entire dataset using the formula: V_adj_ = V_raw_ – (β * eTIV_centered_). For dataset 2, which did not include a limited/no pathology group, the limited/no pathology group used in dataset 1 was reused for eTIV-adjustment. To assess discriminative performance for the structural MRI metrics in antemortem MRI, we calculated receiver operator characteristics (ROC) using the pROC package in R [52] and derived the sensitivity, specificity and corresponding area under the curve (AUC). Higher AUC values indicate greater ability to differentiate the groups, with AUC > 0.8 typically considered “good” and AUC > 0.9 typically considered “excellent” [53]. Based on the ROC models, optimal cut-off values for each metric were calculated using Youden’s Index, which identifies the point of maximum distance from the chance line giving equal weight to sensitivity and specificity (i.e., maxima for sensitivity + specificity – 1).

We next applied these cut-off values (derived from dataset 1) in dataset 2 and evaluated their discriminatory performance when applied to a new dataset. Due to observations detailed in the results section, we then performed ROC analyses pooling data from both antemortem datasets 1 and 2. Note that these ROC analyses were conducted post-hoc following the results of the initial derivation–validation approach conducted separately in datasets 1 and 2. For our main models applied in the pooled dataset, we included age as a covariate. This allowed us to estimate an age-adjusted decision threshold, specifically assessing how the optimal classification threshold changes with each year of age. We derived the rate of change in the optimal threshold per year (Δthreshold/Δage) by examining the relative influence of age and the main predictor within the model. Confidence intervals for this estimated relationship were calculated using the Delta Method [54].

Following this, we ran several sensitivity analyses to test the robustness of the discriminatory performance derived in the pooled dataset. Specifically, we evaluated the change to AUC values associated with the different cohorts (CNDR or ADNI) by including it as a covariate. We then performed stratified analyses dividing the HS-A group into those with none-to-low ADNC and intermediate-to-high ADNC. Finally, we performed stratified analyses dividing the HS-A group into those with FTLD-TDP and LATE-NC.

## Results

### Demographic characteristics

In our postmortem dataset, both HS-A cases and limited/no pathology cases were on average older than those with ADNC (see Table 1). HS-A cases in the postmortem dataset had higher scores on the Clinical Dementia Rating (CDR) and lower scores on the Mini Mental State Examination (MMSE) compared to both ADNC and limited/no pathology cases, indicating greater cognitive impairment. Only one HS-A case in the postmortem dataset had intermediate to high ADNC. The limited/no pathology cases had lower average years of education (but a considerably larger standard deviation).

In antemortem dataset 1, but not antemortem dataset 2, cases with HS-A had higher CDR scores and lower MMSE than ADNC and limited/no pathology cases. Scores on MMSE were lower and scores for CDR higher for both the ADNC and HS-A groups in antemortem dataset 1 compared to antemortem dataset 2. The composition of the HS-A group was slightly different in the two antemortem datasets. In antemortem dataset 1 three out of four cases with HS-A had a neuropathologic diagnosis of frontotemporal lobar degeneration with TDP-43 inclusions (FTLD-TDP) and one case had LATE neuropathologic change (LATE-NC), whereas in antemortem dataset 2 six of our seven cases with HS-A had a neuropathological diagnosis of LATE-NC and one of FTLD-TDP. Moreover, none of the HS-A cases had comorbid intermediate-high ADNC in antemortem dataset 1, whereas five out of seven HS-A cases had intermediate to high ADNC in antemortem dataset 2. Finally, cases in antemortem dataset 1 were qualitatively slightly younger, included a lower proportion of men and lower MMSE scores compared to those in antemortem dataset 2.

### Characterization of the MRI signature of HS-A

All HS-A cases evaluated displayed severe neuronal loss and associated cortical thinning encompassing the full length of the hippocampus (see Nissl-stained sections in Fig. 1). In postmortem MRI the corresponding thinning of the grey matter tissue is clearly visible across the majority of subiculum and CA1, with particularly prominent thinning closer to the CA1/subiculum junction (blue arrows in Fig. 1, Postmortem T_2_ MRI). Cases with HS-A also displayed hypointense gray matter signal (approximating white matter signal in T_2_ MRI) which tended to be even more selective to the CA1/subiculum junction (green arrows in Fig. 1, Postmortem T_2_ MRI). These qualitative markers were consistent across both HS-A cases with comorbid FTLD-TDP and LATE-NC (visual comparison of both groups available in Supplementary Figure S4).

In antemortem MRI, thinning of the CA1 and subiculum regions can be clearly observed in most T_2_ images (green arrows, Antemortem T_2_ MRI). However, in T_1_ MRI the limited resolution precludes clear visibility of focal atrophy patterns, particularly since SRLM, which delineates the inner boundaries of the hippocampus, is poorly visualized in T_1_ MRI. Intensity changes also show almost no visibility on antemortem MRI (both T_1_ and T_2_ MRI), likely due to a combination of limited resolution, the time difference between antemortem and postmortem MRI, and the focal nature of hypointensity pattern observed in postmortem MRI.

Taken together, these observations suggest that measures based on thickness at the CA1/subiculum junction could be particularly valuable biomarkers for the detection of HS-A on antemortem MRI. Although intensity changes were not visually prominent, their potential as biomarkers warranted further investigation through quantitative analysis, where subtle but meaningful differences might still be detected. Additionally, since we observed cortical thinning covering the entire length of the hippocampus, we reasoned that global volume estimates from automated segmentations might be sufficiently sensitive for HS-A classification.

### Group comparisons of postmortem MRI metrics across HS-A, ADNC and limited/no pathology cases

**Fig. 2:**
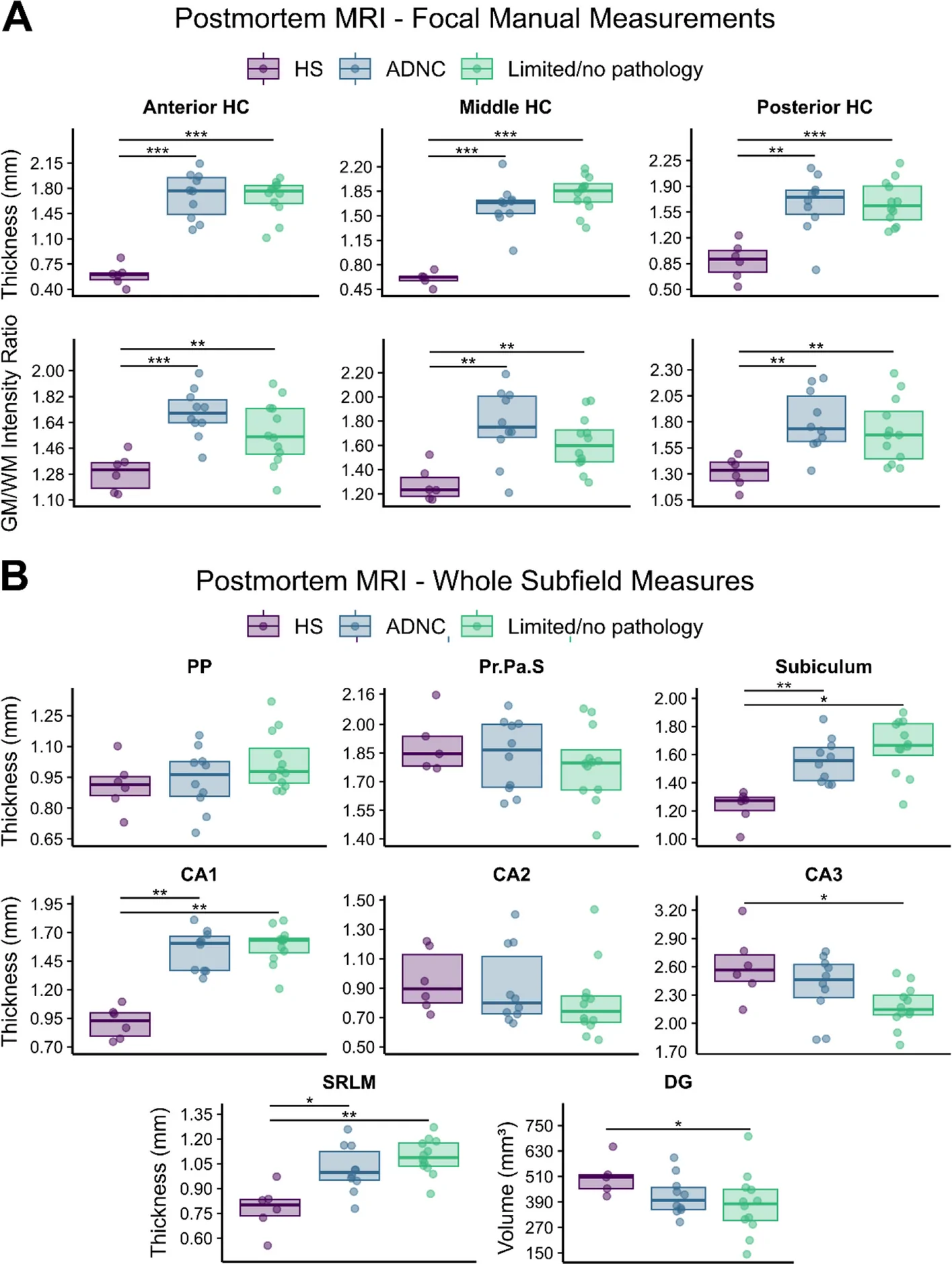
Comparison of MRI metrics across HS-A, ADNC and limited/no pathology cases in postmortem MRI. **A** Thickness and GM/WM intensity ratios manually extracted at select locations in the anterior, middle and posterior hippocampus. **B** Global subfield thickness measures (and volume for dentate gyrus) derived using an in-house developed postmortem automated segmentation algorithm. HS-A: Hippocampal sclerosis of aging; ADNC: Alzheimer’s Disease neuropathologic change; PP: Perforant pathway (white matter of subiculum); Pr.Pa.S: Pre- and Para-subiculum; CA1-3: Cornu Ammonis 1–3; SRLM: Stratum radiatum lacunosum moleculare; DG: dentate gyrus. Comparisons made using nonparametric testing (Mann Whitney U Test) and adjusted for multiple comparison using FDR. ***: p_adj_ < 0.001, **: p_adj_ < 0.01, *: p_adj_ < 0.05

For the manually extracted measurements, we found that HS-A cases had significantly thinner CA1/subiculum in the anterior hippocampus (HS-A vs ADNC: rank-biserial *r* = 1.00, *p*_*adj*_ = 0.002, HS-A vs Limited/no pathology: rank-biserial *r* = 1.00, *p*_*adj*_ = 0.002), middle hippocampus (HS-A vs ADNC: rank-biserial *r* = 1.00, *p*_*adj*_ = 0.002; HS-A vs Limited/no pathology: rank-biserial *r* = 1.00, *p*_*adj*_ = 0.002) and posterior hippocampus (HS-A vs ADNC: rank-biserial *r* = 0.88, *p*_*adj*_ = 0.006; HS-A vs Limited/no pathology: rank-biserial *r* = 1.00, *p*_*adj*_ = 0.002). In the anterior and middle hippocampus (but not the posterior), CA1/subiculum in HS-A was so thin that there was no overlap with the two reference groups. For GM/WM intensity ratios, HS-A cases had significantly lower values, meaning more hypointense signal in the grey matter (approximating white matter), compared to ADNC and limited/no pathology cases in the anterior hippocampus (HS-A vs ADNC: rank-biserial *r* = 0.97, *p*_*adj*_ = 0.012; HS-A vs Limited/no pathology: rank-biserial *r* = 0.75, *p*_*adj*_ < 0.013), middle hippocampus (HS-A vs ADNC: rank-biserial *r* = 0.83, *p*_*adj*_ = 0.012; HS-A vs Limited/no pathology: rank-biserial *r* = 0.81, *p*_*adj*_ = 0.012) and posterior hippocampus (HS-A vs ADNC: rank-biserial *r* = 0.90, *p*_*adj*_ = 0.012; HS-A vs Limited/no pathology: rank-biserial *r* = 0.75, *p*_*adj*_ = 0.013). Boxplots showing the comparisons are available in Fig. 2 and full statistical documentation in Supplementary Table S1. Similar patterns were found for HS-A cases with comorbid FTLD-TDP and LATE-NC (Supplementary Figure S5).

For global measures of subfields thickness (volume for dentate gyrus) obtained using an automated segmentation approach, we further corroborated these findings showing thinner CA1 (HS-A vs ADNC: rank-biserial *r* = 1.00, *p*_*adj*_ = 0.005; HS-A vs Limited/no pathology: rank-biserial *r* = 1.00, *p*_*adj*_ = 0.005) and subiculum (HS-A vs ADNC: rank-biserial *r* = 1.00, *p*_*adj*_ = 0.005; HS-A vs Limited/no pathology: rank-biserial *r* = 0.89, *p*_*adj*_ = 0.009) in HS-A compared to the two reference groups. For both, the thickness observed in HS-A was so low that there was almost no overlap between HS-A and the two reference groups. HS-A cases also had significantly thinner SRLM than both reference groups (HS-A vs ADNC: rank-biserial *r* = 0.80, *p*_*adj*_ = 0.025; HS-A vs Limited/no pathology: rank-biserial *r* = 0.97, *p*_*adj*_ = 0.005). Differences found here are not surprising given that SRLM houses the white matter layer of the CA region [55]. Across remaining subfields we found no significant differences, highlighting the focal nature of HS-A-related atrophy. Boxplots showing the comparisons are available in Fig. 2 and full statistical documentation in Supplementary Table S1. Similar patterns were found for HS-A cases with comorbid FTLD-TDP and LATE-NC (see Supplementary Figure S5).

### Group comparisons of antemortem MRI metrics across HS-A, ADNC and limited/no pathology cases

**Fig. 3:**
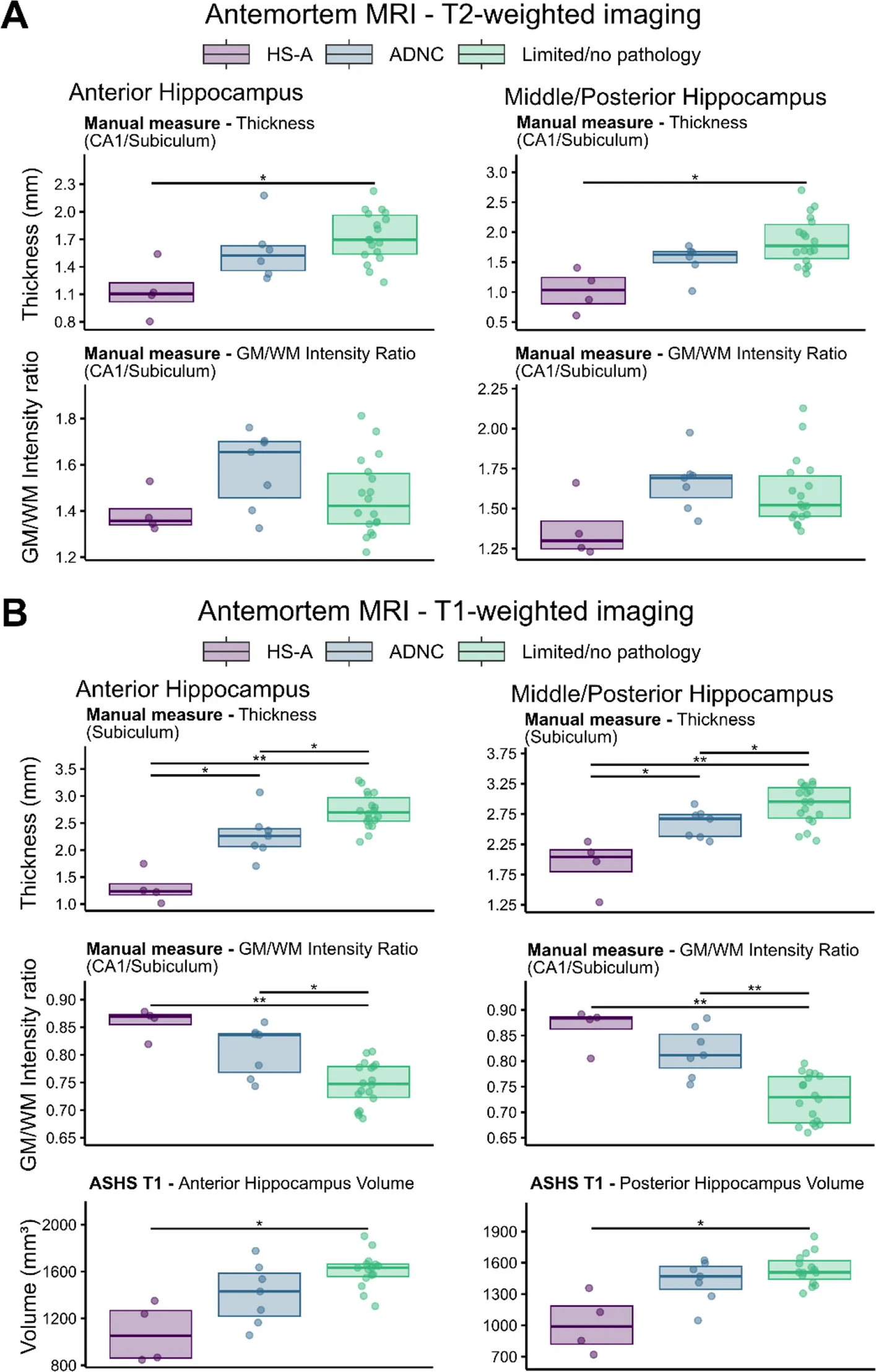
Comparison of MRI metrics across HS-A, ADNC and limited/no pathology cases in antemortem MRI. **A** Metrics using T2 MRI. **B** Metrics using T1 MRI. HS-A: Hippocampal sclerosis of aging; ADNC: Alzheimer’s Disease neuropathologic change; CA1: Cornu Ammonis 1. Comparisons made using nonparametric tests (Mann Whitney U Test) and adjusted for multiple comparison using FDR. ***: p_adj_ < 0.001, **: p_adj_ < 0.01, *: p_adj_ < 0.05

Comparisons presented here are based on antemortem MRI dataset 1. Boxplot visualizations of relevant comparisons discussed below are available in Fig. 3. For manually extracted measurements in T_2_ MRI, we found that the CA1/subiculum junction was significantly thinner in HS-A cases compared to limited/no pathology in both anterior hippocampus (rank-biserial *r* = 0.86, *p*_*adj*_ = 0.028) and middle/posterior hippocampus (rank-biserial *r* = 0.94, *p*_*adj*_ = 0.026). CA1/subiculum was also significantly thinner in HS-A compared to ADNC in the middle/posterior hippocampus; however, this did not survive FDR correction. For GM/WM intensity ratios in T_2_ MRI, we found that HS-A cases had lower values, meaning more hypointense signal in the grey matter (approximating white matter) compared to the ADNC group in the middle/posterior hippocampus, however this comparison did not survive FDR correction. See Supplementary Table S2 for full statistical documentation.

For manually extracted thickness measurements in T_1_ MRI, we found that HS-A cases exhibited significantly thinner subiculum compared to both ADNC cases and limited/no pathology cases in the anterior hippocampus (HS-A vs ADNC: rank-biserial *r* = 0.93, *p*_*adj*_ = 0.022; HS-A vs Limited/no pathology: rank-biserial *r* = 1.00, *p*_*adj*_ = 0.08) and middle/posterior hippocampus (HS-A vs ADNC: rank-biserial *r* = 1.00, *p*_*adj*_ = 0.016; HS-A vs Limited/no pathology: rank-biserial *r* = 1.00, *p*_*adj*_ = 0.08). In terms of GM/WM intensity ratios in T_1_ MRI, we found that HS-A cases showed significantly higher values – indicating more hyperintense signal, similar to white matter – compared to limited/no pathology cases in both the anterior hippocampus (rank-biserial *r* = 1.00, *p*_*adj*_ = 0.005) and middle/posterior hippocampus (rank-biserial *r* = 1.00, *p*_*adj*_ = 0.005). No statistically significant differences were observed comparing HS-A and ADNC. See Supplementary Table S2 for full statistical documentation.

For automated global subfield measures obtained from T_2_ MRI scans, we found statistically significantly or marginally significantly smaller volumes for almost all subfields of the hippocampus in HS-A cases compared to both reference groups. Only the subiculum did not differ significantly between HS-A and ADNC (presented in Supplementary Table S5 and Supplementary Figure S7). This was unexpected given the restricted atrophy pattern observed on postmortem MRI. However, these findings were likely due to insufficient segmentation quality among HS-A cases because of the degree of atrophy and overall image quality. Specifically, across HS-A cases there was a general tendency for ASHS to undersegment the hippocampus and dentate gyrus specifically, as well as a tendency to oversegment the CA1 and subiculum regions (example given in Supplementary Figure S8). For that reason, we believe the results on automated segmentations of T_2_ MRI are less valid than the segmentations obtained from the postmortem MRI. See Supplementary Table S2 for full statistical documentation.

For global hippocampal measures obtained with ASHS-T_1_, we found significantly smaller volumes in HS-A compared to limited/no pathology cases in the anterior hippocampus (rank-biserial *r* = 0.97, *p*_*adj*_ = 0.013) and in posterior hippocampus (rank-biserial *r* = 0.97, *p*_*adj*_ = 0.013). HS-A compared to ADNC had significantly smaller posterior but not anterior hippocampal volumes, however this comparison did not survive FDR correction. See Supplementary Table S2 for full statistical documentation.

Due to the single HS-A case with LATE-NC for antemortem dataset 1, no direct comparison of these subtypes was conducted here. However, given the partial overlap between the antemortem dataset 1 and postmortem dataset, correlations between postmortem and antemortem MRI metrics were calculated, which are available in Supplementary Figure S6. Overall, the postmortem and antemortem MRI measurements of similar regions were significantly correlated. The antemortem MRI metrics were consistently larger than the postmortem MRI metrics, potentially due to the larger voxel size in the antemortem MRI but also slight difference in the measurement locations. Note that the GM/WM intensity ratios were negatively correlated because of the different sequences used postmortem and antemortem (T_1_ versus T_2_).

### Evaluation of discriminative performance of antemortem MRI metrics

#### Antemortem MRI dataset 1

We next compared discriminative performance of the different metrics using ROC analyses and corresponding performance metrics. For this analysis, we focused on the differentiation of HS-A and ADNC as this provides the most diagnostic value. Given the previously described validity issue pertaining to the T_2_ automated measures, these were not included here. We also did not include the measures of GM/WM intensity ratios as they revealed smaller group differences compared to the thickness/volume measures. Across the remaining metrics, AUC values indicated good to excellent performance, ranging from AUC = 0.82 at the lowest and AUC = 1.00 at the highest, with manual metrics generally outperforming automated approaches (see Table 2). Sensitivity ranged from 0.75 to 1.00 across all evaluated metrics, while specificity ranged from 0.57 to 1.00. Table 2 summarizes the performance of all evaluated metrics.

**Table 2:** Discriminatory performance (HS-A vs. ADNC) in antemortem dataset 1 and for both antemortem datasets combined

Metrics	AUC (95% CI)	Optimal Cutoff	Sensitivity	Specificity	PPV	NPV
*Dataset 1*						
Manual T_1_ – Focal Thickness						
Anterior Hippocampus	0.96 (0.87, 1.00)	1.83 mm	1.00	0.86	0.80	1.00
Middle/Posterior Hippocampus	1.00 (1.00, 1.00)	2.30 mm	1.00	1.00	1.00	1.00
Manual T_2_ – Focal Thickness						
Anterior Hippocampus	0.88 (0.61, 1.00)	1.20 mm	0.75	1.00	1.00	0.86
Middle/Posterior Hippocampus	0.92 (0.73, 1.00)	1.43 mm	1.00	0.83	0.80	1.00
ASHS T_1_—Global Volume						
Anterior Hippocampus	0.86 (0.58, 1.00)	1388 mm^3^	1.00	0.57	0.57	1.00
Posterior Hippocampus	0.93 (0.77, 1.00)	1382 mm^3^	1.00	0.71	0.67	1.00
*Pooled analysis adjusted for age (dataset 1 + dataset 2)*						
Manual T_1_ – Focal Thickness						
Anterior Hippocampus	0.87 (0.74, 0.99)	1.45 mm	0.80	0.88	0.73	0.92
Middle/Posterior Hippocampus	0.80 (0.65, 0.96)	2.081 mm	0.60	0.88	0.67	0.85
ASHS T_1_—Global Volume						
Anterior Hippocampus	0.96 (0.91, 1.00)	1017 mm^3^	0.90	0.92	0.82	0.96
Posterior Hippocampus	0.94 (0.88, 1.00)	1150 mm^3^	1.00	0.84	0.71	1.00
Whole Hippocampus	0.98 (0.94, 1.00)	2326 mm^3^	1.00	0.88	0.77	1.00

*AUC *Area under Curve, *CI* Confidence interval, *PPV *Positive Predictive Value, *NPV *Negative Predictive value, *ASHS *Automatic Segmentation of Hippocampal Subfields

#### Antemortem MRI dataset 2

Next, we applied optimal threshold values derived in dataset 1 to the metrics calculated in dataset 2 (group comparison statistics available in Supplementary Table S6). Note that for dataset 2, only T_1_ MRI was available. In the second data set, there was a large decrease in performance (results available in Supplementary Table S7). While sensitivity remained high, ranging from 0.83 to 1.00, specificity was consistently low, ranging from 0.33 to 0.50.

There are several reasons one might expect the performance drop when applying cut-offs derived from dataset 1 to dataset 2. Dataset 1 has a small sample size which can lead to unstable thresholds using Youden’s Index. Additionally, the two HS-A groups differ in several important ways, including a majority of dataset 2 cases being older, having a larger proportion of HS-A cases with comorbid AD, and with LATE-NC instead of FTLD-TDP (see Table 1). For these reasons, we ran additional analyses pooling the two antemortem datasets (N_HS-A_ = 10, N_AD_ = 25), allowing us to evaluate the contribution of these factors to classification accuracy.

#### Evaluation of discriminative performance in pooled dataset

**Fig. 4:**
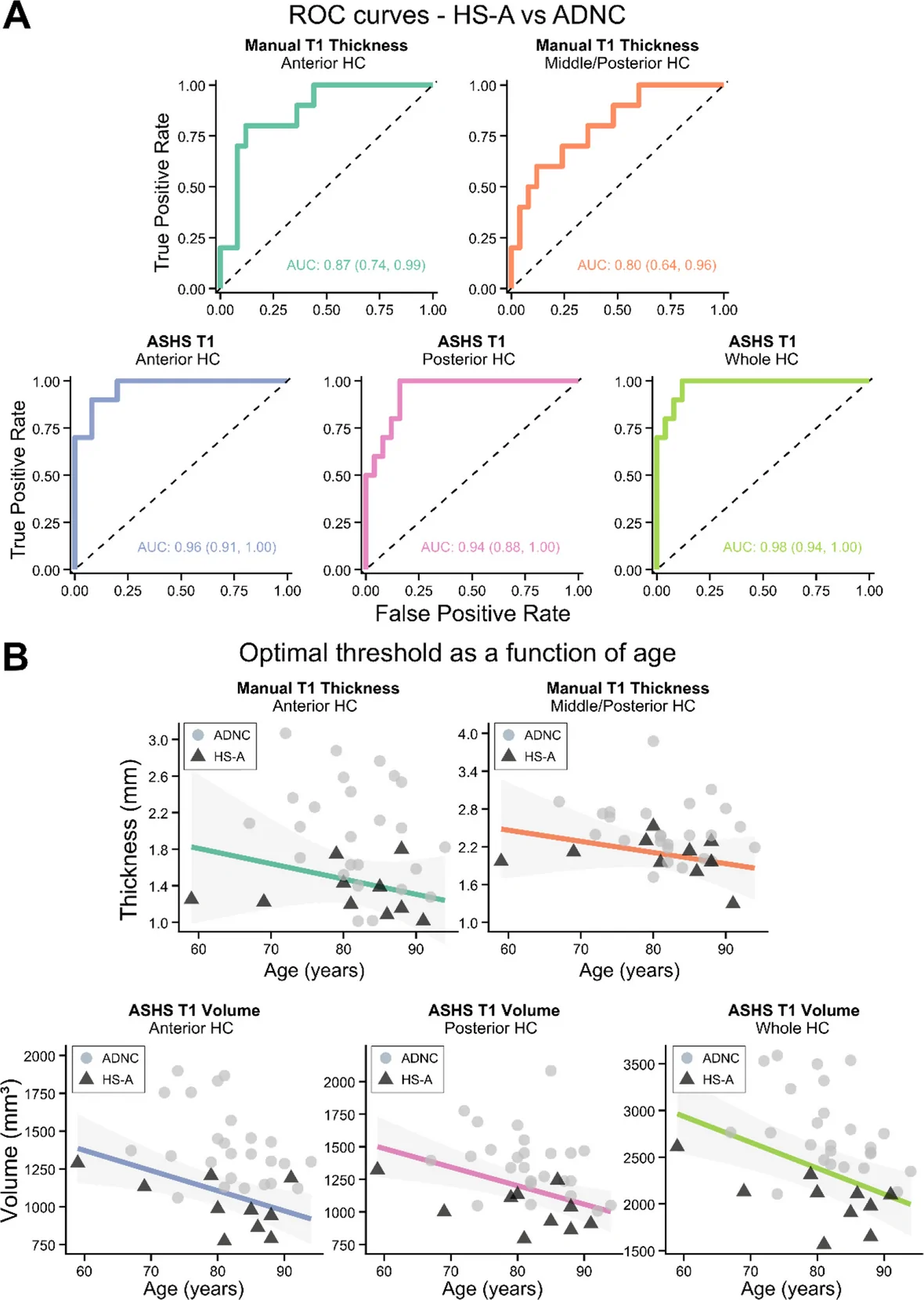
**A** Age-adjusted ROC-curves assessing discriminability of HS-A and ADNC cases across different metrics in data pooled from antemortem dataset 1 and dataset 2. **B** Derived optimal thresholds extracted using Youden’s Index varied as a function of age (regression lines), with confidence intervals extracted using the Delta Method. Black triangles = HS-A, Grey circles = ADNC. AUC: Area under Curve; ASHS: Automatic Segmentation of Hippocampal Subfields

Figure 4 presents ROC-curves and corresponding AUC values for each metric in the pooled dataset. Age was included as covariate in the models, as it is a potential confounder and would be available in a clinical setting. Here we found that automated approaches outperformed our manual measures displaying excellent discriminatory power in both anterior hippocampus (AUC = 0.96, 95% CI = [0.91, 1.00]) and posterior hippocampus (AUC = 0.94, 95% CI = [0.88, 1.00]) (see Fig. 4A and Table 2). Given the high AUC values for both anterior and posterior hippocampus volumes, we also calculated the performance of whole hippocampal volumes, which also demonstrated excellent performance (AUC = 0.98, 95% CI = [0.94, 1.00]).

Including age as a covariate allowed estimation of age-adjusted decision thresholds, showing how the optimal classification cut-off shifts with age. Across all metrics, the estimated threshold decreases with higher age, as highlighted in Fig. 4B.

#### Sensitivity analysis of discriminative performance in pooled dataset

For sensitivity analyses, we next performed stratified ROC analyses dividing the HS-A group into those with none-to-low ADNC and intermediate-to-high ADNC, as well as into those with FTLD-TDP and LATE-NC. We also computed ROC models including the specific cohort as a covariate. Across these iterations, changes to AUC values typically ranged from −0.04 to 0.06, with the exception of manual measurement of thickness in middle/posterior hippocampus, where classification performance made a more substantial increase from AUC = 0.80 to AUC = 0.92 among FTLD-TDP in isolation, and a slight drop among LATE-NC cases from AUC = 0.80 to AUC = 0.77. Given the small sample size, these changes should be interpreted with caution.

## Discussion

To date, assessment of neuronal loss relative to tau burden at autopsy remains the sole established way of identifying HS-A. Developing in vivo biomarkers for the identification of HS-A in living patients would greatly improve diagnosis and prognosis in memory clinics. Aiming to evaluate the potential utility of MRI as a diagnostic tool in the classification of HS-A [19, 22–24], we first characterized MRI signatures of HS-A utilizing ultra-high resolution postmortem imaging. Here, cortical thinning could be seen clearly across hippocampal subfields CA1 and subiculum and typically, but not as consistently, darkening of the same gray matter (approximating the signal of the white matter) could be observed in a more focal location near the CA1/subiculum junction. Next, we demonstrated that thickness measures of CA1 and subiculum, obtained either manually or with an automated approach, could differentiate HS-A from ADNC and limited/no pathology in postmortem MRI, with more subtle differences found for measures of GM/WM signal intensity. We then aimed to translate these findings to antemortem MRI and showed promising differentiation in antemortem dataset 1 but a substantial drop in specificity applying the T_1_ imaging thresholds in antemortem dataset 2. Given limited sample size and pathological heterogeneity, we pooled the two antemortem datasets for post-hoc analysis. Here, manual measures of thickness had good discrimination performance (AUC > 0.8) separating HS-A from ADNC whereas anterior, posterior and whole hippocampal volumes, obtained with an automated approach from T_1_ images, displayed excellent discriminatory performance (AUC > 0.9).

To our knowledge, this is the first study to investigate HS-A using ultra-high-resolution postmortem MRI with corresponding neuropathological confirmation of HS-A. The availability of same-subject neuropathology and ultra-high-resolution postmortem MRI provided us with a unique opportunity to characterize the MR signatures of HS-A with a high level of detail. Consistent throughout our cases was severe neuronal loss across CA1 and subiculum, which was reflected in cortical thinning in postmortem MRI. This, we found, was most clearly visible at a location corresponding to the CA1/subiculum junction, in line with previous observations that this area might be an early locus of HS-A pathology (e.g., [56]). Interestingly, the CA1/subiculum junction is a region that receives innervation from the amygdala [57], a region early affected by TDP-43, which, in turn, is hypothesized to be a precursor to HS-A [6]. Atrophy in HS-A for the most part covered the full length of the hippocampus, however compared to ADNC and limited/no pathology cases the difference was most prominent in anterior portion of hippocampus. This corroborates the previously observed anterior–posterior gradient for HS-A atrophy [19, 56]. Given that HS-A is seen as end-stage TDP-43 pathology [6], it also aligns with an anterior–posterior gradient observed for TDP-43 pathology within the hippocampus [58]. In the present study, atrophy in HS-A as seen in postmortem MRI was evident in both CA1 and subiculum. However, no significant differences were found in CA2, CA3 or dentate gyrus compared to ADNC and limited/no pathology, demonstrating a very selective pattern of atrophy typical of HS-A [22, 23]. These findings can serve as a reference for future in vivo studies interrogating hippocampal subfield atrophy patterns.

Less consistently we observed signal intensity changes of the grey matter tissue (approximating white matter) in the target region. This tended to be more localized and include only a narrow portion close to the CA1 and subiculum junction, the hypothesized earliest locus of HS-A [4, 56]. Fitting these observations, HS-A cases had significantly lower GM/WM intensity ratios in the target region compared to ADNC and limited/no pathology cases. We suspect the focal nature of intensity changes might relate to known changes in glia cells during the progression of HS-A, exhibiting hypercellularity in earlier stages of HS-A progression [56]. Increased signal intensity of grey matter tissue in T_2_ MRI due to gliosis (i.e. becoming more distinct from white matter) is a well-documented phenomenon in hippocampal sclerosis in the context of temporal epilepsy [59–61]. This might explain the observation that signal intensity changes are most clearly visible in the earliest locus, where such glial reactivity has presumably subsided. The hypothesized lack of glial reactivity combined with severe neuronal loss may there result in an MRI signal that is more hypointense, resembling white matter.

Next, we evaluated whether similar metrics could be applied in antemortem MRI. Specifically, in antemortem dataset 1, we evaluated the discriminatory performance of metrics applied in both isotropic T_1_ scans and anisotropic T_2_ scans with high coronal in-plane resolution. Here, measures of thickness and hippocampal volume outperformed GM/WM signal intensity. For replication in antemortem dataset 2, we therefore focused on measures of thickness and volume, specifically targeting T_1_ metrics due to the replication dataset having no T_2_ images available. We initially tested applying specific cut-off values derived from antemortem dataset 1 in dataset 2 but here observed a substantial drop in performance, primarily due to reduced specificity. This likely reflects model instability arising from the limited sample size, as well as differences in age and neuropathological diagnoses between HS-A cases across the two datasets.

To address this, we conducted post-hoc analyses in a pooled dataset combining both antemortem dataset 1 and dataset 2. In this analysis, we show that the optimal threshold decreases with higher age. Although postmortem analyses revealed specific atrophy patterns within the hippocampus in HS-A, our findings suggest that diagnostic differentiation may be achieved through global hippocampal volumetric markers that demonstrate a quantitatively greater degree of atrophy in this group. While global hippocampal atrophy is not specific to HS-A, the severity atrophy may be. This mirrors earlier reports of HS-A cases having smaller hippocampal volumes than ADNC and controls [20, 23, 24] and the complete loss of neurons in CA1 and subiculum, not reported in other neuropathological diagnoses. Additionally, the utility of relatively simple hippocampal volume measures from standard T_1_ MRI scans is promising given the widespread clinical availability. Global hippocampal estimates can be obtained using publicly available automated approaches such as ASHS, FreeSurfer, or MAGeT Brain [41, 57, 58]. However, further investigation is needed to validate the metrics in the context of each software used.

Although manual metrics were expected to outperform global hippocampal volumes by targeting the most affected regions, they ultimately performed worse in the pooled analysis. This likely reflects challenges of manual measurement, including limited resolution, severe atrophy, and reliance on subtle changes near the limit of visibility. Poor T_2_ image quality may have further contributed to inconsistencies in results regarding hippocampal subfield volume differences between the postmortem and antemortem group comparisons. Future advances in MR imaging as well as recent advances in deep learning–based image processing, including upsampling approaches, may improve application of granular subfield measurements even in the presence of severe atrophy [56].

The current study assessed the differentiation of HS-A, ADNC and cases with limited or no neuropathology, leaving the question open of how well the applied metrics would differentiate HS-A from other diseases affecting the hippocampus such as LATE-NC without HS-A or combined ADNC and LATE-NC without HS-A. Another important consideration for future investigation is the specific TDP-43 pathology tied to HS-A, as this study included cases with both comorbid FTLD-TDP and LATE-NC. Previous findings have suggested differences between these two patient groups, with more reactive gliosis and a more extensive neuronal loss associated with HS-A in the context of LATE-NC [62]. Qualitatively, we saw little to no difference comparing HS-A cases with FTLD-TDP and LATE-NC in the postmortem dataset. We also conducted sensitivity analyses in our pooled antemortem analysis where classification performance showed little to no difference between HS-A cases with low or intermediate-high ADNC, between HS-A cases with comorbid FTLD-TDP or LATE-NC, or across site. Together, these findings give preliminary support to the utility of MRI metrics in the diagnostic differentiation of HS-A and ADNC across a diverse set of comorbid neuropathological diagnoses.

## Limitations

The combination of a small sample size and pathological heterogeneity of the HS-A group are clear limitations of the current study, rendering the findings of the study preliminary and in need of further validation. At the same time, the pathological heterogeneity allowed us to investigate HS-A across different neuropathological diagnosis. However, this was only possible at a qualitative level. In the antemortem analysis, although we restricted the scan–autopsy interval to five years to increase the likelihood that HS-A was present at the time of scanning, most cases had intervals of several years. As a result, disease progression at the time of MRI likely varied across cases. Additionally, the pooled antemortem analysis should be considered exploratory, as the discrimination performance observed in dataset 1 did not replicate when the derived thresholds were applied to dataset 2. Finally, although diagnostic accuracy was evaluated with adjustment for acquisition site, the limited sample size precluded more fine-grained adjustment for scanner manufacturer and model, where hardware differences may influence volumetric measures [63].

## Conclusion

In this study, we uniquely characterized the MRI signature of neuropathologically verified HS-A using ultra-high resolution postmortem MRI, revealing localized changes in thickness and grey matter voxel intensity that were distinctive of HS-A. With respect to translation to antemortem MRI, our results suggest that measures of whole hippocampal volume or volume of anterior and posterior hippocampus, obtained from standard resolution MRI, are the most sensitive to differentiate HS-A from ADNC, which is promising given that these measures can be relatively easily obtained in a clinical setting. Although these specific metrics need to be further validated in a larger sample comparing HS-A to several other dementia-related diseases, they demonstrate promising potential for future in vivo clinical identification of HS-A using MRI.

## Supplementary Information


Supplementary Material 1.


## Data Availability

Data from HNL and CNDR are not publicly available but can be requested from the specific sites. Data from ADNI is available via the ADNI website.

## Abbreviations

HS-A: Hippocampal Sclerosis of Aging
AD: Alzheimer’s Disease
ADNC: AD neuropathologic change
TDP-43: TAR DNA-binding protein 43
LATE: Limbic-predominant Age-related TDP-43 Encephalopathy
LATE-NC: LATE neuropathologic change
FTLD: Frontotemporal lobar degeneration
MRI: Magnetic Resonance Imaging
ASHS: Automated Segmentation of Hippocampal Subfields
GM: Grey matter
WM: White matter
HNL: Human Neuroanatomy Lab, Albacete, Spain
CNDR: Pennsylvania’s Center for Neurodegenerative Disease Research
ADNI: Alzheimer’s Disease Neuroimaging Initiative Database
CA1-3: Cornu ammonis 1–3
SRLM: Stratum Radiatum Lacunosum Moleculare
DG: Dentate Gyrus
Pr.Pa.S: Pre- and Para-subiculum
PP: Perforant pathway
FDR: False Discovery Rates
AUC: Area Under Curve
ROC: Receiver Operator Characteristics
CDR: Clinical Dementia Rating scale
MMSE: Mini Mental State Examination
CI: Confidence Intervals
