# Supplementary material for: Characterizing the MRI signature of hippocampal sclerosis of aging
Source: Alzheimers Res Ther. 2026 Jun 22;18:153. doi: 10.1186/s13195-026-02118-0 (PMC13289427; doi:10.1186/s13195-026-02118-0)
Supplement: Supplementary file 1 [file 13195_2026_2118_MOESM1_ESM.docx]

## Table S1. Case-level information for the postmortem dataset (HNL/CNDR).

| **Case** | **Age** **autopsy** | **Sex** | **Site** | **Hemisphere used** | **ADNC** **stage** | **TDP**  **CA/SUB** | **TDP**  **ERC** | **TDP** **DG** | **TDP** **AMY** | **Primary NP diagnosis** |
| --- | --- | --- | --- | --- | --- | --- | --- | --- | --- | --- |
| HS-A 1 | >89 | Male | CNDR | Right | 1 | 2 | 3 | 2 | 3 | FTLD-TDP |
| HS-A 2 | 83 | Female | CNDR | Right | 0 | 2 | 3 | 1 | 2 | LATE-NC |
| HS-A 3 | 72 | Male | CNDR | Left | 1 | 2 | 2 | 1 | 0.5 | FTLD-TDP |
| HS-A 4 | 83 | Male | CNDR | Left | 1 | 0.5 | 3 | 2 | 3 | LBD |
| HS-A 5 | 82 | Male | CNDR | Left | 1 | 1 | 3 | 2 | 2 | FTLD-TDP |
| HS-A 6 | >89 | Female | CNDR | Left | 2 | 0 | 1 | 0.5 | 1 | ADNC |
| ADNC 1 | 75 | Male | CNDR | Left | 3 | 0 | 0 | 0 | 0 | ADNC |
| ADNC 2 | 75 | Male | CNDR | Right | 2 | 0 | 0 | 0 | 0 | ADNC |
| ADNC 3 | 88 | Male | CNDR | Right | 2 | 0 | 0 | 0 | 0 | ADNC |
| ADNC 4 | 69 | Male | CNDR | Right | 3 | 0 | 0 | 0 | 0 | ADNC |
| ADNC 5 | 76 | Female | CNDR | Right | 3 | 0 | 0 | 0 | 0 | ADNC |
| ADNC 6 | 84 | Male | CNDR | Left | 2 | 0 | 0 | 0 | 0 | LBD |
| ADNC 7 | 76 | Male | CNDR | Left | 2 | 0 | 0 | 0 | 0 | ADNC |
| ADNC 8 | 74 | Male | CNDR | Left | 2 | 0 | 0 | 0 | 0 | LBD |
| ADNC 9 | 78 | Female | CNDR | Left | 3 | 0 | 0 | 0 | 0 | ADNC |
| ADNC 10 | 74 | Female | CNDR | Right | 2 | 0 | 0 | 0 | 0 | LBD |
| Lim./no path 1 | >89 | Male | CNDR | Right | 1 | 0 | 0 | 0 | 0 | ADNC |
| Lim./no path 2 | 85 | Female | CNDR | Right | 1 | 0 | 0 | 0 | 0 | ADNC |
| Lim./no path 3 | 78 | Female | HNL | Left | 1 | 0 | 0 | 0 | 0 | ADNC |
| Lim./no path 4 | 71 | Male | HNL | Right | 1 | 0 | 0 | 0 | 0 | ADNC |
| Lim./no path 5 | >89 | Male | HNL | Right | 1 | 0 | 0 | 0 | 0 | ADNC |
| Lim./no path 6 | >89 | Female | HNL | Right | 1 | 0 | 0 | 0 | 0 | ADNC |
| Lim./no path 7 | >89 | Male | HNL | Left | 1 | 0 | 0 | 0 | 0 | ADNC |
| Lim./no path 8 | 74 | Male | HNL | Right | 1 | 0 | 0 | 0 | 0 | ADNC |
| Lim./no path 9 | 68 | Female | HNL | Left | 1 | 0 | 0 | 0 | 0 | ADNC |
| Lim./no path 10 | 71 | Male | HNL | Left | 1 | 0 | 0 | 0 | 0 | ADNC |
| Lim./no path 11 | 68 | Male | HNL | Left | 0 | 0 | 0 | 0 | 0 | None |
| Lim./no path 12 | 81 | Male | HNL | Right | 1 | 0 | 0 | 0 | 0 | ADNC |

*Note:* TDP variables represent semiquantitative scores rating each region as none (0), rare (0.5), mild (1), moderate (2), or severe (3) for individual lesions. HS-A = Hippocampal Sclerosis of Aging, ADNC = Alzheimer’s Disease Neuropathologic Change, FTLD-TDP = Frontotemporal lobar degeneration with transactive TAR DNA-binding protein 43 (TDP-43) inclusion, LBD = Lewy Body Disease, CNDR = Center for Neurodegenerative Disease Resarch, HNL = Human Neuroanatomy Lab, CA = Cornu Ammonis, SUB = Subiculum, ERC = Entorhinal cortex, DG = Dentate Gyrus, AMY = Amygdala, NP = Neuropathological

^a^ Age above 89 is not specified because it is considered sensitive health data and due to identification risk given sample size.

## Table S2. Case-level information for antemortem dataset 1 (CNDR/ADNI).

| **Case** | **Sex** | **Age  at MRI** | **Age at autopsy** | **Scan-autopsy interval (y)** | **Site** | **Hemisphere used** | **Scanner** | **T2 slice thickness (mm)** | **ADNC stage** | **TDP-43** **AMY** | **TDP-43** **DG** | **TDP-43** **ERC** | **Primary NP diagnosis** |
| --- | --- | --- | --- | --- | --- | --- | --- | --- | --- | --- | --- | --- | --- |
| HS-A 1 | Male | >89 | >89 | 3 | CNDR | Both | Siemens Prisma | 1.2 | 1 | 3 | 2 | 3 | FTLD-TDP |
| HS-A 2 | Female | 79 | 83 | 4 | CNDR | Both | Siemens Skyra | 3 | 0 | 2 | 1 | 3 | LATE-NC |
| HS-A 3 | Male | 69 | 72 | 3 | CNDR | Both | Siemens TrioTrim | 2 | 1 | 0.5 | 1 | 2 | FTLD-TDP |
| HS-A 7 | Female | 59 | 62 | 3 | CNDR | Left | Siemens TrioTrim | 2 | 0 | 2 | 2 | 1 | FTLD-TDP |
| ADNC 1 | Male | 67 | 75 | 8 | CNDR | Right | Siemens TrioTim | 2 | 3 | 0 | 0 | 0 | ADNC |
| ADNC 5 | Female | 74 | 76 | 2 | CNDR | Left | Siemens Prisma | 1.2 | 3 | 0 | 0 | 0 | ADNC |
| ADNC 6 | Male | 81 | 84 | 3 | CNDR | Right | Siemens Skyra | 3 | 2 | 0 | 0 | 0 | LBD |
| ADNC 7 | Male | 74 | 76 | 2 | CNDR | Right | Siemens Skyra | 3 | 2 | 0 | 0 | 0 | ADNC |
| ADNC 8 | Male | 72 | 74 | 2 | CNDR | Right | Siemens Prisma | 1.2 | 2 | 0 | 0 | 0 | LBD |
| ADNC 9 | Female | 76 | 78 | 2 | CNDR | Right | Siemens Prisma | 1.2 | 3 | 0 | 0 | 0 | ADNC |
| ADNC 10 | Female | 73 | 74 | 1 | CNDR | Left | Siemens Prisma Fit | 1.2 | 2 | 0 | 0 | 0 | LBD |
| Lim./no path 13 | Male | 68 | - | - | ADNI | Both | Siemens Prisma Fit | 2 | - | - | - | - | - |
| Lim./no path 14 | Female | 70 | - | - | ADNI | Both | Siemens Verio | 2 | - | - | - | - | - |
| Lim./no path 15 | Female | 70 | - | - | ADNI | Both | Siemens Prisma Fit | 2 | - | - | - | - | - |
| Lim./no path 16 | Male | 72 | - | - | ADNI | Both | Siemens Prisma Fit | 2 | - | - | - | - | - |
| Lim./no path 17 | Male | 76 | - | - | ADNI | Both | Siemens Prisma | 2 | - | - | - | - | - |
| Lim./no path 18 | Female | 83 | - | - | ADNI | Both | Siemens Prisma Fit | 2 | - | - | - | - | - |
| Lim./no path 19 | Female | 80 | - | - | ADNI | Both | GE Discovery MR750 | 2 | - | - | - | - | - |
| Lim./no path 20 | Male | 77 | - | - | ADNI | Both | GE Discovery MR750 | 2 | - | - | - | - | - |
| Lim./no path 21 | Female | 69 | - | - | ADNI | Both | Philips Achieva | 2 | - | - | - | - | - |
| Lim./no path 22 | Female | 72 | - | - | ADNI | Both | Siemens Verio | 2 | - | - | - | - | - |
| Lim./no path 23 | Male | 77 | - | - | ADNI | Both | Siemens Verio | 2 | - | - | - | - | - |
| Lim./no path 24 | Female | 75 | - | - | ADNI | Both | Philips Ingenia | 2 | - | - | - | - | - |
| Lim./no path 25 | Female | 76 | - | - | ADNI | Both | GE Discovery MR750 | 2 | - | - | - | - | - |
| Lim./no path 26 | Female | 68 | - | - | ADNI | Both | GE Discovery MR750 | 2 | - | - | - | - | - |
| Lim./no path 27 | Female | 75 | - | - | ADNI | Both | Philips Achieva dStream | 2 | - | - | - | - | - |
| Lim./no path 28 | Female | 70 | - | - | ADNI | Both | Siemens Verio | 2 | - | - | - | - | - |
| Lim./no path 29 | Female | 79 | - | - | ADNI | Both | Siemens Prisma Fit | 2 | - | - | - | - | - |
| Lim./no path 30 | Female | 68 | - | - | ADNI | Both | Siemens Prisma Fit | 2 | - | - | - | - | - |

*Note:* TDP variables represent semiquantitative scores rating each region as none (0), rare (0.5), mild (1), moderate (2), or severe (3) for individual lesions. In cases where both hemispheres were used, neuropathological confirmation of HS-A was confirmed in both hemispheres. HS-A = Hippocampal Sclerosis of Aging, ADNC = Alzheimer’s Disease Neuropathologic Change, FTLD-TDP = Frontotemporal lobar degeneration with transactive TAR DNA-binding protein 43 (TDP-43) inclusion, LBD = Lewy Body Disease, CNDR = Center for Neurodegenerative Disease Resarch, ADNI = Alzheimer’s Disease Neuroimaging Initiative, CA=Cornu Ammonis, SUB = Subiculum, ERC = Entorhinal cortex, DG = Dentate Gyrus, AMY = Amygdala, NP = Neuropathological

^a^ Age above 89 is not specified because it is considered sensitive health data and due to identification risk given sample size.

## Table S3. Case-level information for antemortem dataset 2 (ADNI).

| **Case** | **Sex** | **Age at MRI**^a^ | **Age at autopsy** | **Scan-autopsy interval (y)** | **Hemisphere used** | **Scanner** | **Arteriosclerosis** | **ADNC**  **stage** | **TDP-43**  **AMY** | **TDP-43**  **HC** | **TDP-43** **ERC** | **Primary NP diagnosis**^b^ |
| --- | --- | --- | --- | --- | --- | --- | --- | --- | --- | --- | --- | --- |
| HS-A 8 | Male | 80 | 80 | 0 | Right | Siemens Sonata | 1 | 1 | 1 | 1 | 1 | - |
| HS-A 9 | Male | <=89 | >89 | 5 | Left | Siemens TrioTim | 3 | 3 | 1 | 1 | 1 | - |
| HS-A 10 | Male | 88 | 89 | 1 | Left | Philips Achieva | 1 | 2 | 1 | 1 | 1 | - |
| HS-A 11 | Male | 81 | 82 | 1 | Left | Siemens SymphonyTim | 1 | 1 | 0 | 1 | 1 | - |
| HS-A 12 | Male | 86 | 88 | 2 | Left | GE Signa Excite | 3 | 3 | 1 | 1 | 1 | - |
| HS-A 13 | Male | 85 | >89 | 5 | Left | GE Signa HDx | 2 | 3 | 1 | 0 | 1 | - |
| HS-A 14 | Male | 88 | >89 | 2 | Left | Siemens Symphony | 1 | 3 | 1 | 1 | 1 | - |
| ADNC 11 | Female | 82 | 84 | 2 | Left | GE Discovery MR750 | 1 | 3 | 0 | 0 | 0 | - |
| ADNC 12 | Male | >89 | >89 | 4 | Left | Siemens Sonata | 2 | 3 | 0 | 0 | 0 | - |
| ADNC 13 | Female | 87 | >89 | 4 | Left | Siemens TrioTim | 1 | 3 | 0 | 0 | 0 | - |
| ADNC 14 | Male | >89 | >89 | 1 | Left | Siemens SymphonyTim | 2 | 2 | 0 | 0 | 0 | - |
| ADNC 15 | Female | 80 | 85 | 5 | Right | Siemens SymphonyTim | 2 | 3 | 0 | 0 | 0 | - |
| ADNC 16 | Male | 82 | 83 | 1 | Left | Siemens Trio | 1 | 3 | 0 | 0 | 0 | - |
| ADNC 17 | Male | 88 | 89 | 1 | Left | GE Signa HDxt | 2 | 3 | 0 | 0 | 0 | - |
| ADNC 18 | Male | 84 | 85 | 1 | Left | GE Discovery MR750 | 1 | 3 | 0 | 0 | 0 | - |
| ADNC 19 | Male | 81 | 84 | 3 | Left | GE Signa HDx | 2 | 3 | 0 | 0 | 0 | - |
| ADNC 20 | Female | 79 | 80 | 1 | Right | Siemens Sonata | 1 | 3 | 0 | 0 | 0 | - |
| ADNC 21 | Male | 85 | 90 | 5 | Left | Siemens TrioTim | 1 | 3 | 0 | 0 | 0 | - |
| ADNC 22 | Male | 88 | 90 | 2 | Left | Siemens Symphony | 1 | 3 | 0 | 0 | 0 | - |
| ADNC 23 | Female | 85 | 88 | 3 | Left | Siemens Verio | 2 | 2 | 0 | 0 | 0 | - |
| ADNC 24 | Male | 85 | 86 | 1 | Left | Philips Intera | 3 | 3 | 0 | 0 | 0 | - |
| ADNC 25 | Male | 82 | 85 | 3 | Left | GE Discovery MR750 | 1 | 3 | 0 | 0 | 0 | - |
| ADNC 26 | Male | >89 | >89 | 2 | Left | GE Signa HDxt | 2 | 2 | 0 | 0 | 0 | - |
| ADNC 27 | Male | 81 | 82 | 1 | Left | GE Discovery MR750 | 1 | 3 | 0 | 0 | 0 | - |
| ADNC 28 | Male | 80 | 81 | 1 | Left | Siemens Symphony | 1 | 2 | 0 | 0 | 0 | - |
| ADNC 29 | Male | 88 | 89 | 1 | Left | Siemens TrioTim | 1 | 2 | 0 | 0 | 0 | - |

*Note:* TDP-43 variables represent binary scores for presence or absence of TDP-43 lesions. ADNC = Alzheimer’s Disease Neuropathologic Change, TDP-43 = TAR DNA-binding protein 43, ERC = Entorhinal cortex, HC = Hippocampus, AMY = Amygdala, NP = Neuropathology, Arteriolosclerosis: 0=0 None; 1=1 Mild; 2=2 Moderate; 3=3 Severe.

^a^ Age above 89 is not specified because it is considered sensitive health data and due to identification risk given sample size.

^b ^Primary neuropathological diagnoses variables are currently archived in the neuropathology data of ADNI at the time of writing.

## Table S4. Statistical comparisons of all metrics evaluated in postmortem MRI between HS-A, ADNC and control cases.

|  | HS-A vs ADNC  (N_HS-A _= 6, N_ADNC_ = 10) |  |  |  | HS-A vs Control  (N_HS-A _= 6, N_CONTROL_ = 12) |  |  |  |
| --- | --- | --- | --- | --- | --- | --- | --- | --- |
| Measure | *U* | *r* | P | P-adj | *U* | *r* | P | P-adj |
| *Manual measurements* |  |  |  |  |  |  |  |  |
| Focal thickness – Anterior hippocampus | 0 | 1.00 | **0.001** | **0.002** | 0 | 1.00 | **0.001** | **0.002** |
| Focal thickness – Middle hippocampus | 0 | 1.00 | **0.001** | **0.002** | 0 | 1.00 | **0.001** | **0.002** |
| Focal thickness – Posterior hippocampus | 4 | 0.87 | **0.006** | **0.006** | 0 | 1.00 | **0.001** | **0.002** |
| Intensity Ratio – Anterior hippocampus | 1 | 0.97 | **0.002** | **0.012** | 9 | 0.75 | **0.013** | **0.013** |
| Intensity Ratio – Middle hippocampus | 5 | 0.83 | **0.008** | **0.012** | 7 | 0.81 | **0.008** | **0.012** |
| Intensity Ratio – Posterior hippocampus | 3 | 0.90 | **0.004** | **0.012** | 9 | 0.75 | **0.013** | **0.013** |
| *Automated measurements* |  |  |  |  |  |  |  |  |
| Thickness – CA1 |  | 1.00 | 0.001 | 0.005 | 0 | 1.00 | 0.001 | 0.005 |
| Thickness – CA2 | 36 | 0.20 | 0.551 | 0.643 | 53 | 0.47 | 0.122 | 0.214 |
| Thickness – CA3 | 40 | 0.33 | 0.303 | 0.424 | 63 | 0.75 | 0.013 | 0.026 |
| Thickness – Subiculum | 0 | 1.00 | **0.001** | **0.005** | 4 | 0.89 | 0.003 | 0.009 |
| Thickness – Pr.Pa-subiculum ^a^ | 29 | 0.16 | 0.668 | 0.668 | 39 | 0.30 | 0.370 | 0.471 |
| Thickness – SRLM | 66 | 0.80 | **0.011** | **0.025** | 1 | 0.97 | 0.001 | 0.005 |
| Thickness – PP | 25 | 0.17 | 0.625 | 0.668 | 20 | 0.44 | 0.147 | 0.228 |
| Volumes – CA1 ^a^ | 1 | 0.96 | **0.004** | **0.028** | 5 | 0.83 | **0.01** | **0.034** |
| Volumes – CA2 ^a^ | 23 | 0.08 | 0.854 | 0.854 | 36 | 0.20 | 0.562 | 0.656 |
| Volumes – CA3 ^a^ | 23 | 0.08 | 0.854 | 0.854 | 52 | 0.73 | **0.023** | 0.054 |
| Volumes – Subiculum ^a^ | 1 | 0.96 | **0.004** | **0.028** | 5 | 0.83 | **0.01** | **0.034** |
| Volumes – DG ^a^ | 38 | 0.52 | 0.126 | 0.176 | 49 | 0.63 | 0.051 | 0.08 |
| Volumes – SRLM ^a^ | 14 | 0.44 | 0.198 | 0.253 | 9 | 0.70 | **0.031** | 0.054 |
| Volumes – PP ^a^ | 4 | 0.84 | **0.012** | **0.034** | 9 | 0.70 | **0.031** | 0.054 |

^a^ N_HS-A _ = 5

Comparisons made using nonparametric Mann Whitney U Test (MWU) and adjusted for multiple comparison using false discovery rate (FDR) across all measures of the same type (e.g., all manual measures of thickness) and group comparisons. Statistically significant effects are marked in bold. *r* = rank-biserial correlation coefficient.  N_HS _= 6, N_AD_ = 10, N_CNTRL_ = 12. HS-A: Hippocampal sclerosis of aging; ADNC: Alzheimer’s Disease neuropathologic change. CA1-3: Cornu Ammonis 1-3; Pr.Pa.S: Pre- and Para-subiculum; SRLM: Stratum radiatum lacunosum moleculare; PP: Perforant pathway (white matter of subiculum); DG: Dentate gyrus.

## Table S5. Statistical comparisons of all metrics evaluated in antemortem MRI between HS-A, ADNC and control cases in dataset 1

|  |  | HS-A vs ADNC  (N_HS-A _= 4, N_ADNC_ = 7) |  |  |  | HS-A vs Control  (N_HS-A _= 4, N_CONTROL_ = 18) |  |  |  |
| --- | --- | --- | --- | --- | --- | --- | --- | --- | --- |
| Measure | Sequence | *U* | *r* | P | P-adj | *U* | *r* | P | P-adj |
| *Manual measurements* |  |  |  |  |  |  |  |  |  |
| Thickness – Anterior hippocampus | T_1_ | 1 | 0.93 | **0.018** | **0.022** | 0 | 1.00 | **0.003** | **0.008** |
| Thickness – Middle/posterior hippocampus | T_1_ | 0 | 1.00 | **0.011** | **0.016** | 0 | 1.00 | **0.003** | **0.008** |
| Intensity Ratio – Anterior hippocampus | T_1_ | 24 | 0.71 | 0.073 | 0.087 | 72 | 1.00 | **0.003** | **0.005** |
| Intensity Ratio – Posterior hippocampus | T_1_ | 22 | 0.57 | 0.156 | 0.156 | 72 | 1.00 | **0.003** | **0.005** |
| Thickness – Anterior hippocampus^b^ | T_2_ | 3 | 0.75 | 0.070 | 0.105 | 5 | 0.86 | **0.009** | **0.028** |
| Thickness – Posterior hippocampus^b^ | T_2_ | 2 | 0.83 | **0.043** | 0.086 | 2 | 0.94 | **0.004** | **0.026** |
| Intensity Ratio – Anterior hippocampus | T_2_ | 5 | 0.64 | 0.108 | 0.163 | 28 | 0.22 | 0.523 | 0.523 |
| Intensity Ratio – Posterior hippocampus | T_2_ | 3 | 0.79 | **0.047** | 0.163 | 13 | 0.64 | 0.055 | 0.163 |
| *Automated measurements* |  |  |  |  |  |  |  |  |  |
| ASHS volume – Anterior hippocampus^a^ | T_1_ | 5 | 0.64 | 0.108 | 0.130 | 1 | 0.97 | **0.004** | **0.013** |
| ASHS volume – Posterior hippocampus^a^ | T_1_ | 3 | 0.79 | **0.047** | 0.094 | 1 | 0.97 | **0.004** | **0.013** |
| ASHS volume – CA1^c^ | T_2_ | 3 | 0.79 | **0.047** | 0.079 | 0 | 1.00 | **0.003** | **0.022** |
| ASHS volume – CA2^c^ | T_2_ | 1 | 0.93 | **0.018** | **0.039** | 2 | 0.94 | **0.005** | **0.027** |
| ASHS volume – CA3^c^ | T_2_ | 2 | 0.86 | **0.03** | 0.056 | 3 | 0.91 | **0.007** | **0.027** |
| ASHS volume – SUB^c^ | T_2_ | 7 | 0.50 | 0.219 | 0.235 | 5 | 0.84 | **0.012** | **0.037** |
| ASHS volume – DG^c^ | T_2_ | 1 | 0.93 | **0.018** | **0.039** | 0 | 1.00 | **0.003** | **0.022** |

^a ^N_CONTROL _= 15

^b ^N_ADNC_ = 6

^c ^N_CONTROL_ = 16

Comparisons made using nonparametric Mann Whitney U Test (MWU) and adjusted for multiple comparison using false discovery rate (FDR) across all measures of the same type (e.g., all manual measures of thickness) and group comparisons. Statistically significant effects are marked in bold. *r* = rank-biserial correlation coefficient. HS-A: Hippocampal sclerosis of aging; ADNC: Alzheimer’s Disease neuropathologic change. HC: Hippocampus; CA1-3: Cornu Ammonis 1-3; SUB: Subiculum; DG: Dentate gyrus. ASHS: Automatic Segmentation of Hippocampal Subfields.

## Table S6. Statistical comparisons of all metrics evaluated in antemortem MRI between HS-A and ADNC cases in dataset 2

|  |  |  |  | HS-A vs ADNC |  |  |  |
| --- | --- | --- | --- | --- | --- | --- | --- |
| Measure | Sequence | N_HS_ | N_AD_ | *U* | *r* | P | P-adj |
| Thickness - Anterior hippocampus | T_1_ | 7 | 19 | 29 | 0.564 | **0.032** | **0.065** |
| Thickness - Posterior hippocampus | T_1_ | 6 | 19 | 34 | 0.404 | 0.152 | 0.152 |
| ASHS volume – Anterior hippocampus | T_1_ | 6 | 18 | 0 | 1.00 | **<0.001** | **0.001** |
| ASHS volume – Posterior hippocampus | T_1_ | 6 | 18 | 6 | 0.889 | **0.002** | **0.002** |

Comparisons made using nonparametric testing (Mann Whitney U Test) and adjusted for multiple comparison using false discovery rate (FDR) across all measures of the same type (e.g., all manual measures of thickness) and group comparisons. *r* = rank-biserial correlation coefficient. HS-A: Hippocampal sclerosis of aging; ADNC: Alzheimer’s Disease neuropathologic change. CA1-3: Cornu Ammonis 1-3; SUB: Subiculum; DG: Dentate gyrus. ASHS: Automatic Segmentation of Hippocampal Subfields.

## Table S7. Discriminative performance of manual and automated metrics in antemortem T_1_ MRI for HS-A and ADNC cases applying cut-offs, derived from antemortem dataset 1, in dataset 2

| Metrics | Optimal Cutoff | Sensitivity | Specificity | PPV | NPV |
| --- | --- | --- | --- | --- | --- |
| Manual T_1_ Thickness Anterior | 1.83 mm | 1.00 | 0.44 | 0.38 | 1.00 |
| Manual T_1_ Thickness Posterior | 2.30 mm | 0.83 | 0.50 | 0.36 | 0.90 |
| ASHS T_1_ Volume Anterior | 1388 mm^3^ | 1.00 | 0.33 | 0.33 | 1.00 |
| ASHS T_1_ Volume Posterior | 1382 mm^3^ | 1.00 | 0.39 | 0.35 | 1.00 |

*Note:* Cut-off values were derived and tested on volumes adjusted for total intracranial volume (TIV). ASHS: Automatic Segmentation of Hippocampal Subfields. PPV: Positive Predictive Value; NPV: Negative Predictive Value.

**Figure S1.** Flowcharts depicting the exclusion and inclusion of cases in the postmortem dataset and antemortem dataset 1. HS-A = Hippocampal Sclerosis of Aging, ADNC = Alzheimer’s Disease neuropathologic change, TDP-43 = TAR DNA-binding protein 43, HNL = Human Neuroanatomy Lab, CNDR = Center for Neurodegenerative Disease Research.

**Figure S2.** Flowcharts depicting the exclusion and inclusion of cases in antemortem dataset 2 from ADNI, and the limited/no pathology reference group from ADNI. HS-A = Hippocampal Sclerosis of Aging, ADNC = Alzheimer’s Disease neuropathologic change, TDP-43 = TAR DNA-binding protein 43, ADNI = Alzheimer’s Disease Neuroimaging Initiative.

**Figure S3.** Examples of manual extraction across the anterior, middle and posterior hippocampus (HC). Top row depicts measurements performed in postmortem MRI, middle row antemortem T_2_ MRI, and bottom row antemortem T_1_ MRI. Left images for anterior HC, middle HC and posterior HC depict extraction of grey matter and white matter voxel intensities from which a ratio is derived (GM/WM intensity). Right images depict extractions of thickness made at three separate locations within select slices. In antemortem imaging, samples were drawn only for anterior hippocampus and for a portion corresponding to the middle of hippocampus.

**Figure S4.** Postmortem MRI for HS-A cases with comorbid LATE-NC and FTLD-TDP. Cases depicted: 1 = Male, 83; 2 = Female, 93; 3 = Male, 94; 4 = Female, 83; 5 = Male, 82; 6 = Male, 72. All cases display clear cortical thinning centered on the border of CA1 and subiculum (green arrow mark approximate center location). No clear difference is noticeable between the HS-A LATE-NC and FTLD-TDP cases.

**Figure S5.** Qualitative comparison of HS-A with LATE-NC vs HS-A with FTLD-TDP in postmortem MRI, including cases with ADNC and controls as reference. **A:** Thickness and GM/WM intensity ratios manually extracted at select locations in anterior, middle and posterior hippocampus. Similar thinning and hypointense grey matter signal can be observed for both HS-A groups. **B**: Global subfield thickness measures (and volume for dentate gyrus) derived using an in-house developed postmortem automated segmentation algorithm. Qualitatively smaller thickness values are observed for both groups in CA1, subiculum and SRLM, but not for other regions. HS-A: Hippocampal sclerosis of aging; ADNC: Alzheimer’s Disease neuropathologic change; PP: Perforant pathway (white matter of subiculum); Pr.Pa.S: Pre- and Para-subiculum; CA1-3: Cornu Ammonis 1-3; SRLM: Stratum radiatum lacunosum moleculare; DG: dentate gyrus.

**Figure S6.** Pairwise Spearman Correlations comparing postmortem MRI metrics and antemortem MRI metrics. Note that intensity ratio in antemortem metrics is derived from a T1-weighted sequence whereas the postmortem metric utilizes a T2-weighted sequences, providing different contrasts. HC: Hippocampus; CA1: Cornu Ammonis 1; GM: Grey matter; WM: White matter; ADNC: Alzheimer’s Disease Neuropathologic Change; HS-A: Hippocampal Sclerosis of Aging.

**Figure S7.** Comparison of hippocampal subfield volumes among cases with HS-A, ADNC and controls in antemortem T_2_ MRI (antemortem dataset 1). Comparisons made using nonparametric testing (Mann Whitney U Test) and adjusted for multiple comparison using FDR across all measures of the same type (e.g., all manual measures of thickness) and group comparisons. ***: p_adj_ < 0.001, **: p_adj_ < 0.01, *: p_adj_ < 0.05. CA1-3: Cornu Ammonis 1-3; DG: dentate gyrus; SUB: Subiculum

**Figure S8**. Examples of ASHS segmentation error in T_2_ MRI in HS-A. Examples from anterior and posterior hippocampus (HC) are sampled from two different cases. Left: ASHS segmentations. Right: Marked borders in ASHS segmentation (red) and manually placed at the correct location (yellow). Images are drawn from different cases. Across cases, there was a clear tendency to oversegment cornu ammonis 1 (CA1) and subiculum as indicated by the difference between ASHS segmentation border and manually placed border. Additionally, there was a tendency to undersegment the hippocampus overall, particularly the superior border.
